# Two-stage lot quality assurance sampling framework for monitoring and evaluation of neglected tropical diseases, allowing for imperfect diagnostics and spatial heterogeneity

**DOI:** 10.1371/journal.pntd.0010353

**Published:** 2022-04-08

**Authors:** Adama Kazienga, Luc E. Coffeng, Sake J. de Vlas, Bruno Levecke

**Affiliations:** 1 Department of Translational Physiology, Infectiology and Public Health, Ghent University, Merelbeke, Belgium; 2 Department of Public Health, Erasmus MC, University Medical Center Rotterdam, Rotterdam, The Netherlands; Emory University, UNITED STATES

## Abstract

**Background:**

Monitoring and evaluation (M&E) is a key component of large-scale neglected tropical diseases (NTD) control programs. Diagnostic tests deployed in these M&E surveys are often imperfect, and it remains unclear how this affects the population-based program decision-making.

**Methodology:**

We developed a 2-stage lot quality assurance sampling (LQAS) framework for decision-making that allows for both imperfect diagnostics and spatial heterogeneity of infections. We applied the framework to M&E of soil-transmitted helminth control programs as a case study. For this, we explored the impact of the diagnostic performance (sensitivity and specificity), spatial heterogeneity (intra-cluster correlation), and survey design on program decision-making around the prevalence decisions thresholds recommended by WHO (2%, 10%, 20% and 50%) and the associated total survey costs.

**Principal findings:**

The survey design currently recommended by WHO (5 clusters and 50 subjects per cluster) may lead to incorrect program decisions around the 2% and 10% prevalence thresholds, even when perfect diagnostic tests are deployed. To reduce the risk of incorrect decisions around the 2% prevalence threshold, including more clusters (≥10) and deploying highly specific diagnostic methods (≥98%) are the most-cost saving strategies when spatial heterogeneity is moderate-to-high (intra-cluster correlation >0.017). The higher cost and lower throughput of improved diagnostic tests are compensated by lower required sample sizes, though only when the cost per test is <6.50 *US*$ and sample throughput is ≥3 per hour.

**Conclusion/Significance:**

Our framework provides a means to assess and update M&E guidelines and guide product development choices for NTD. Using soil-transmitted helminths as a case study, we show that current M&E guidelines may severely fall short, particularly in low-endemic and post-control settings. Furthermore, specificity rather than sensitivity is a critical parameter to consider. When the geographical distribution of an NTD within a district is highly heterogeneous, sampling more clusters (≥10) may be required.

## Author summary

Periodic follow-up surveys for monitoring and evaluation (M&E) are an important aspect of large-scale neglected tropical diseases (NTD) programs. They are critical to measure progress and determine whether continuing or scaling down interventions is justified. In absence of better alternatives, imperfect diagnostic tests are often deployed in these surveys. Yet, little is known how this affects the population-based program decision-making. We expanded an existing survey design framework that allows for imperfect diagnostic tests and applied it to M&E of intestinal worms. It is a synonymous of soil-transmitted helminths control programs as a case study. In addition, we assessed the trade-off between cost per test, sample throughput and diagnostic performance of tests. We demonstrated that the current M&E guidelines may lead to incorrect program decisions, even when perfect diagnostic methods are deployed. To reduce the risk of incorrect decisions, including more disease-clusters and deploying highly specific diagnostic methods revealed to be the most cost-saving strategies. On the other hand, the higher cost and lower throughput of improved diagnostic tests can be compensated by lower required sample sizes. In conclusion, our framework can contribute to developing more evidence-based guidelines and choices in diagnostic product development for M&E of NTD control programs.

## Introduction

Neglected tropical diseases (NTD) are a diverse group of 20 parasitic, bacterial, and viral diseases, several of which are zoonotic, food or vector-borne in nature [1–3]. Worldwide, they affect more than 1.5 billion of the world’s population, but disproportionately impact the most impoverished communities in tropical countries [3], particularly those on the African continent [1]. Enormous progress has been made so far in controlling NTD, yet significant challenges remain such as a lack of improved diagnostic tests, new interventions, and monitoring and evaluation (M&E) strategies. As a response to this, WHO has recently published its new road map, aiming (i) to reduce the number of people requiring interventions against NTDs by 90%, (ii) to reduce the global disease burden by 75%, (iii) to eliminate at least one NTD in 100 countries, and (iv) to eradicate two NTD by the end of 2030 [4].

To reach these ambitious 2030 targets, periodic follow-up surveys to measure progress and determine whether scaling down or stopping the interventions is justified, so-called M&E, are important aspects of these large-scale NTD programs. To track progress towards achieving the 2030 targets for NTD, WHO developed and published M&E guidelines for 15 of the 20 NTD [4]. Generally, these M&E guidelines involve recommendations for (*i*) which diagnostic test to use, (*ii*) which survey design to use (e.g., number of subjects and number of clusters), and (*iii*) the corresponding decision rules for continuing or reducing the frequency (or intensity) of interventions. However, the diagnostic tests deployed in these M&E surveys are often imperfect, and it remains unclear how this affects decision-making at the population level. For example, Gass (2020) recently urged for a shift in paradigm from sensitivity to specificity and a holistic approach to developing WHO M&E guidelines for population-based interventions, aligning survey designs and decision rules to the deployed diagnostic test [5].

In a first attempt to gain insight into this complex interplay between diagnostic performance, survey designs and decision rules, we previously developed a general framework based on a 1-stage lot quality assurance sampling (LQAS) approach that consider the impact of diagnostic performance at the individual level (sensitivity and specificity), survey design (e.g., number of subjects), and decision rules on the correctness of program decision-making at the population level [6]. We found that specificity rather than sensitivity becomes more important when the program approaches the endgame. In addition, the study found that the requirements for both parameters are inversely correlated, resulting in multiple combinations of sensitivity and specificity that allow for reliable decision-making. This study further highlighted that improving diagnostic performance results in smaller sample sizes for the same level of program decision-making. Thus, the additional costs per diagnostic test with improved diagnostic performance can be compensated by lower operational costs in the field. These findings have been instrumental in defining the required diagnostic performance of tests deployed in M&E of control programs targeting soil-transmitted helminthiasis [6]. However, an important limitation of this framework is the assumption that all tested subjects originate from the same cluster (e.g., community/school). As such, spatial heterogeneity of infections across clusters was ignored. Also, a detailed cost assessment was not included (e.g., operational costs to collect and screen samples, cost per test and the sample throughput). This is important as improved diagnostic tests might allow decisions to be based on fewer samples, which would compensate for the increased cost per test and reduced sample throughput.

In the present study, we developed a 2-stage LQAS framework for decision-making that allows for both imperfect diagnostics and spatial heterogeneity of infections. Second, we applied the framework using M&E of soil-transmitted helminth (STH) control programs as a case study. For this, we explored the impact of diagnostic performance (sensitivity and specificity), spatial heterogeneity (intra-cluster correlation), and survey design (number of clusters and number of subjects per cluster) on the correctness of program decision-making based on WHO-recommended thresholds, as well as the associated total survey costs. In addition, we evaluated to what extent an increased cost per test and a reduced sample throughput might be compensated by lower sample size requirements when using improved diagnostic tests.

## Methodology

### Development of a 2-stage LQAS framework for population-based decision-making using imperfect diagnostic tests

#### General concepts of the framework

**Fig 1:**
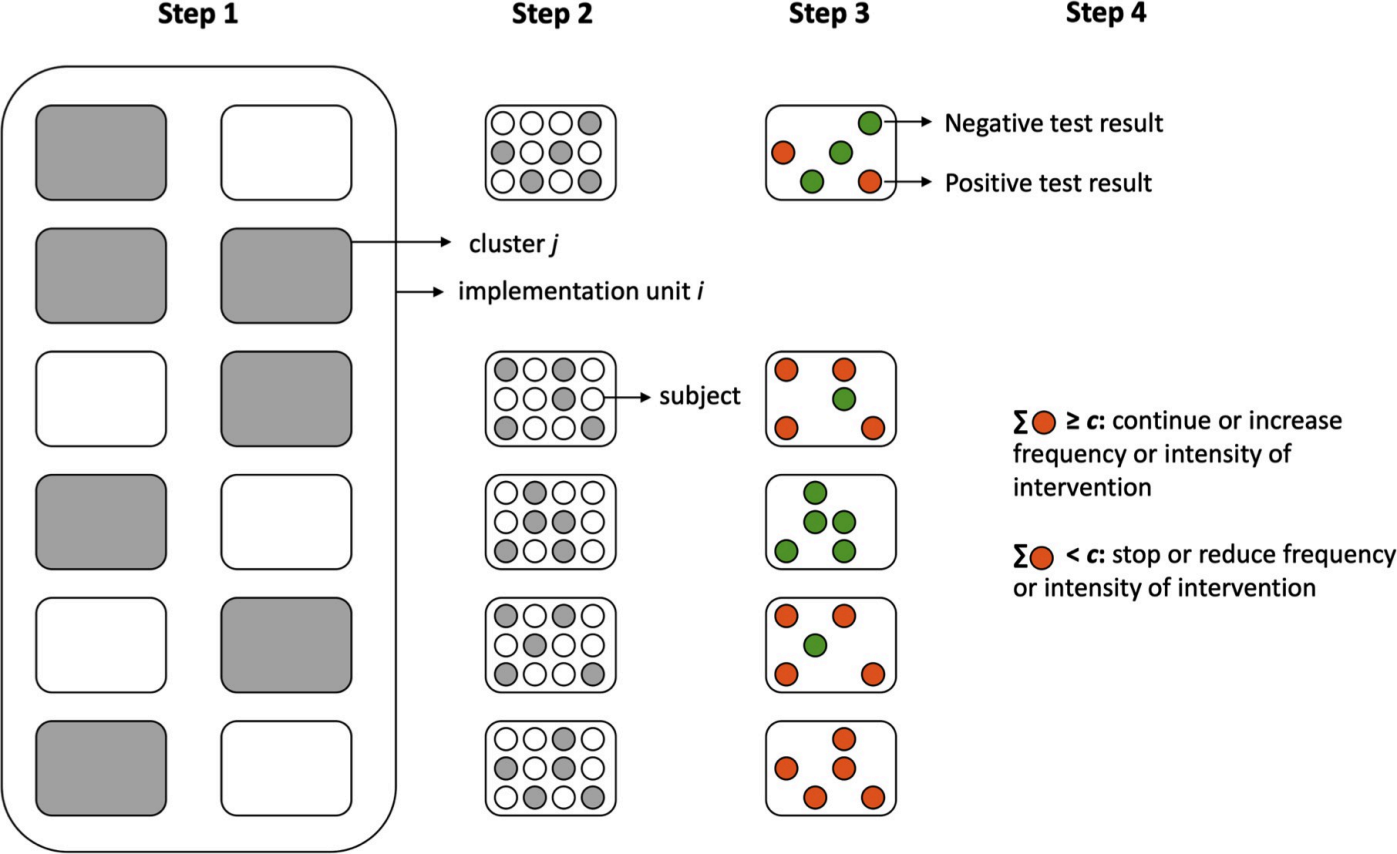
The different steps within the 2-stage LQAS framework for population-based decision-making using an imperfect test.

The 2-stage LQAS framework for population-based decision-making using imperfect diagnostic tests consists of four steps (**Fig 1**). In the **first step**, *n*_*clust*_ clusters (e.g., schools or communities) are randomly selected from a given implementation unit *i* (e.g., a geographical area where preventive chemotherapy (PC) is administered). In the **second step**, *n*_*sub*_ subjects are randomly selected within each cluster *j*. In the **third step**, all *n*_*tot*_ ( = *n*_*clust*_∙*n*_*sub*_) subjects are screened using an imperfect diagnostic test *D* that has a sensitivity *se*_*d*_ and a specificity *sp*_*d*_. In the fourth and **final step**, the program decisions for unit *i* are made based on the number of positive test results (*X*_*i*+_). The frequency (or intensity) of the intervention will be reduced (e.g., scale-down from 6-monthly to annual PC) in case *X*_*i*+_ is less than some decision cut-off *c*. In contrast, when *X*_*i*+_≥*c*, the frequency or intensity of the intervention remains unchanged or will even be increased. Note that *X*_*i*+_ includes both true and false positive test results. Also, the framework allows for the spatial heterogeneity, the so-called intra-cluster correlation *ρ*_*i*_. It is a measure of the extent to which positive test results are clustered within clusters of an implementation unit *i*, and as such, is a measure of how prevalences vary between clusters. The intra-cluster correlation can take on values between zero and one, with higher values representing more clustering of positive test results (i.e., higher geographical heterogeneity in infection levels).

Ultimately, we wish to determine the survey design (*n*_*clust*_ and *n*_*sub*_) and the decision cut-off *c* that allows for an acceptable risk of making incorrect program decision-making when an imperfect diagnostic test *D* is deployed in a unit *i* with intra-cluster correlation *ρ*_*i*_. In other words, we should aim to minimize the probability that interventions are implemented at a frequency or intensity higher than officially necessary based on the true underlying prevalence (i.e., when true prevalence *π*_*i*_ is under the program decision threshold *T*) as this would lead to a waste of both time and resources. Simultaneously, we should aim to minimize the probability that interventions are scaled down prematurely (i.e., when *π*_*i*_≥*T*), which would lead to a preventable increase in infection and morbidity. Here, we assume that the target prevalence threshold *T* is defined in terms of the true prevalence *π*_*i*_ (not as measured by some imperfect diagnostic test). We further assume that the value of *T* is appropriate for scaling interventions up or down, which may [7] or may not be the case [8–10].

**Fig 2:**
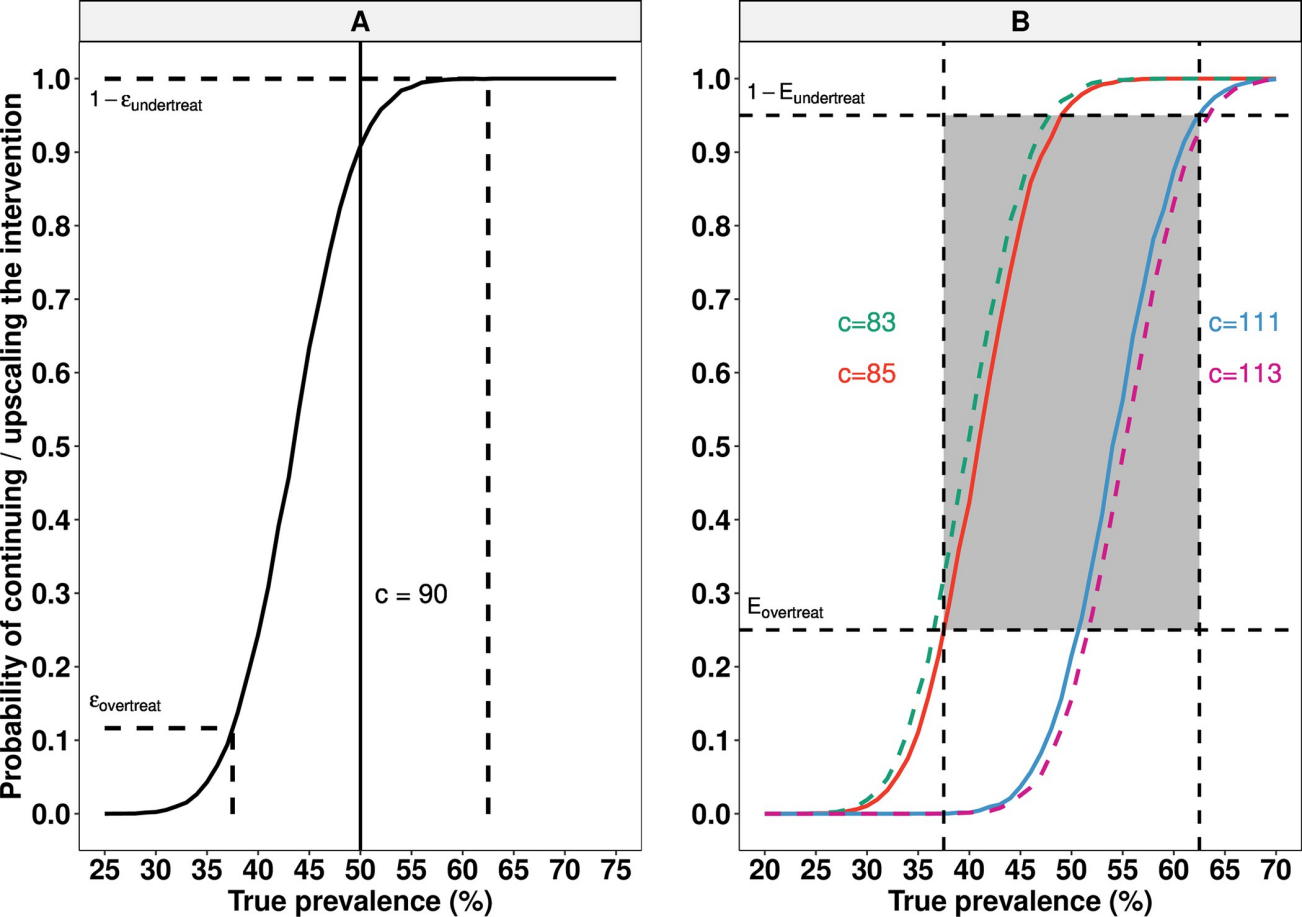
Program decision-making process based on 2-stage LQAS framework allowing for an imperfect diagnostic test. Panel A illustrates the risk of making a wrong program decision (***ε***_***overtreat***_ and ***ε***_***undertreat***_) for a given true prevalence ***π***_***i***_ and decision cut-off ***c*** = **90** was applied. The vertical straight line indicates the program prevalence threshold ***T*** = **50%**, whereas the horizontal dotted lines represent the ***ε***_***overtreat***_ and ***ε***_***undertreat***_ for ***π***_***i***_ = **37.5**% and ***π***_***i***_ = **62.5**%, respectively. Panel B represents the decision cut-off ***c*** for a choice of grey zone width (lower limit (***LL***) = 37.5% and upper limit (***UL***) = 62.5%) and acceptable risk levels (***E***_***overtreat***_ = **25**% and ***ε***_***undertreat***_ = **5**%) for a particular of range of ***π***_***i***_. The solid sigmoid curves (in red and blue) indicate the values of ***c*** that satisfy the conditions $\varepsilon_{overtreat}(\pi_{i}=LL)\leq E_{overtreat}$ and $\varepsilon_{undertreat}(\pi_{i}=UL)\leq E_{undertreat}$, whereas the dotted sigmoid curves (green and purple) do not satisfy the conditions mentioned above. All graphs are based on the same theoretical diagnostic test ***D*** (***se***_***d***_ = **80**% and ***sp***_***d***_ = **98**%), survey design (***n***_***clust***_ = **5** and ***n***_***sub***_ = **50**), an intra-cluster correlation ***ρ***_***i***_ = **0.02** and 10,000 Monte Carlo simulations.

**Fig 2A** illustrates the program decision-making process based on LQAS, using a toy example. It describes the probability of continuing or scaling up the frequency or intensity of the intervention as a function of the true underlying prevalence *π*_*i*_ at the implementation unit level. For this, we assumed that a theoretical diagnostic method *D* was deployed (sensitivity *se*_*d*_ = 80% and specificity *sp*_*d*_ = 98%) to screen *n*_*clust*_ = 5 clusters and *n*_*sub*_ = 50 subjects per cluster. For illustrative purposes, the LQAS decision cut-off was set at *c* = 90 (in a sample of *n*_*tot*_ = *n*_*clust*_∙*n*_*sub*_ = 250). Given a program decision threshold *T* = 50% (vertical straight line), we can now deduce the error probability of unnecessarily continuing or upscaling the intervention at a frequency or intensity that is greater than needed (*ε*_*overtreat*_) when true prevalence *π*_*i*_<*T*, and the error probability of prematurely reducing the frequency or intensity of interventions (*ε*_*undertreat*_) when true prevalence *π*_*i*_≥*T*. These error probabilities can be considered to be community-level analogues of one minus the negative predictive value and one minus the positive predicted value [5], as used in recent NTD modelling exercises [8,11]. In **Fig 2B,** we deduce the decision cut-off *c* for which *ε*_*overtreat*_ and *ε*_*undertreat*_ do not exceed acceptable risk levels for arbitrary choices of *π*_*i*_<*T* and *π*_*i*_≥*T*. For this purpose, we define the minimal survey performance in terms of the (un)acceptable risk levels (*ε*_*overtreat*_ = 25% and *ε*_*undertreat*_ = 5%) for a particular range of *π*_*i*_ close to threshold *T* that is defined by a lower (*LL*) and upper limit (*UL*). Together, they form a “grey zone” that dictates the slope of the sigmoid curve (from lower left bottom to top right corner of the grey zone). We then try to find the range of values for the decision cut-off *c* that satisfy the conditions $\varepsilon_{overtreat}(\pi_{i}=LL=37.5\%)\leq E_{overtreat}$ and $\varepsilon_{undertreat}(\pi_{i}=UL=62.5\%)\leq E_{undertreat},$ and we select the value of decision cut-off *c* that offers the lowest attainable risk of *ε*_*undertreat*_ as we considered this the most important risk to mitigate. As can be seen from **Fig 2B**, the solid lines indicate the values of *c* that satisfy the conditions mentioned above (red line: *c* = 85 and blue line: *c* = 111), and we choose the decision cut-off equalled 85, as it offers the lowest *ε*_*undertreat*_ (0.5%) as compared to 111 (*ε*_*undertreat*_ = 4.7%). On the contrary, the dotted lines represent some values of *c* that do not satisfy the conditions (green line: *c* = 83 and purple line: *c* = 113).

### The mathematical backbone of the framework

The mathematical backbone of the 2-stage LQAS population-based decision-making using an imperfect diagnostic test is summarized by the three equations below (**Eq (1)–(3)**). **Tables 1** and **2** describe the definitions of the different parameters and derived variables used in these equations, respectively. **Eq (1)** describes the 2-stage sampling process that results in the total number of positive test results within a cluster. For this, we used a 2-stage beta-binomial model, where the beta-distribution represents the variation in true cluster-level prevalence *π*_*ij*_ within an implementation unit *i*, and the binomial distribution represents the sample variation in the number of positive tests *X*_*ij*_ resulting in cluster *j* and implementation unit *i* when an imperfect diagnostic test *D* with sensitivity *se*_*d*_ and specificity *sp*_*d*_ is applied. The shape parameters *α*_*i*_ and *β*_*i*_, both greater than zero, define the beta distribution, where index *i* indicates that these parameters can be implementation unit specific. For the present study, we parameterized the beta distribution in terms of the expected value $\pi_{i}=\frac{\alpha_{i}}{\alpha_{i}+\beta_{i}}$ and the intra-cluster correlation $\rho_{i}=\frac{1}{a_{i}+\beta_{i}+1}$, where *α*_*i*_ and *β*_*i*_ are the distributions’ two shape parameters. An added benefit of using the intra-cluster correlation as a measure of variation is that it is independent of the mean *π*_*i*_ of the beta distribution, unlike its variance $\sigma_{i}^{2}(=\frac{\alpha_{i}\beta_{i}}{{\left(\alpha_{i}+\beta_{i}\right)}^{2}(a_{i}+\beta_{i}+1)}=\frac{\pi_{i}(1-\pi_{i})\rho_{i}}{\left(\alpha_{i}+\beta_{i}\right)}=\frac{\pi_{i}(1-\pi_{i})\rho_{i}}{\frac{1}{\rho_{i}}-1}=\frac{\pi_{i}(1-\pi_{i})\rho_{i}^{2}}{1-\rho_{i}})$. To illustrate our framework, we considered an intra-cluster correlation *ρ*_*i*_ of 0.02 [12]. The binomial distribution for positivity of individual test results was parameterized in term of $\pi_{ij+}(=se_{d}∙\pi_{ij}+(1-sp_{d})∙(1-\pi_{ij}))$, the expected proportion of positive test results within a cluster *j* from implementation unit *i*, and the number of subjects screened per cluster (*n*_*sub*_):

$$\begin{array}{l} \pi_{ij}∼Βeta(\pi_{i},\rho_{i})\\ \pi_{ij+}=se_{d}∙\pi_{ij}+(1-sp_{d})∙(1-\pi_{ij})\\ X_{ij+}∼Βinom(n_{sub},\pi_{ij+})\\ X_{i+}=\sum_{j}X_{ij+} \end{array}$$ (Eq 1)

**Table 1:** Definitions of the parameters that describe the 2-stage LQAS framework.

Parameters	Definition
*α*_*i*_, *β*_*i*_	Shape parameters of a beta distribution describing the variation in true cluster-level prevalence within an implementation unit *i*
*π*_*i*_	Expected cluster-level true prevalence within implementation unit *i*; $\pi_{i}=\frac{\alpha_{i}}{\alpha_{i}+\beta_{i}}$.
*ρ*_*i*_	Intra-cluster correlation; $\rho_{i}=\frac{1}{\alpha_{i}+\beta_{i}+1}$
*π*_*ij*_	True prevalence in cluster *j* from implementation unit *i*; *π*_*ij*_~Beta(*π*_*i*_, *ρ*_*i*_) or synonymously *π*_*ij*_~Beta(*α*_*i*_, *β*_*i*_).
*se*_*d*_	Sensitivity of an imperfect diagnostic test *D*
*sp*_*d*_	Specificity of an imperfect diagnostic test *D*
*π*_*ij*+_	Probability of a positive test result in cluster *j* from implementation unit $i;\;\pi_{ij+}=se_{d}∙\pi_{ij}+(1-sp_{d})∙(1-\pi_{ij})$.
*n*_*clust*_	Number of clusters randomly selected per implementation unit *i*
*n*_*sub*_	Number of subjects randomly selected within each cluster *j* from implementation unit *i*
*n*_*tot*_	Total number of subjects randomly selected across *n*_*clust*_ clusters from implementation unit *i*; *n*_*tot*_ = *n*_*sub*_∙*n*_*clust*_
*E*_*overtreat*_	Highest allowed probability of falsely continuing or upscaling an intervention within an implementation unit *i*
*E*_*undertreat*_	Highest allowed probability of prematurely stopping or scaling down interventions within an implementation unit *i*
*LL*, *UL*	Lower and upper limits of the grey zone
*T*	Program decision true prevalence threshold

**Table 2:** Definitions of the derived variables that describe the 2-stage LQAS framework.

Variables	Definition
*X*_*ij*+_	The number of positive test results within a sampled cluster *j* within an implementation unit *i*
*X*_*i*+_	Total number of positive test results across all sampled clusters within an implementation unit *i*
*c*	Decision cut-off for the total number of positive cases used to make program decisions within an implementation unit *i*
*ε*_*overtreat*_	Probability of falsely continuing or upscaling an intervention frequency within an implementation unit *i*
*ε*_*undertreat*_	Probability of prematurely reducing interventions within an implementation unit *i*

**Eq (2)** and **Eq (3)** below represent the risks associated with incorrect program decision-making, denoting the parts of the graph line in **Fig 2A** left and right of the prevalence threshold *T*, respectively. Here, **Eq (2)** describes the probability that the interventions are implemented at a frequency or intensity higher than required for the true underlying prevalence when *π*_*i*_<*T*. Vice versa, **Eq 3** describes the probability that interventions are prematurely stopped or scaled down when *π*_*i*_≥*T*:

$$\varepsilon_{overtreat}=P(X_{i+}\geq c|\pi_{i}<T,\rho_{i},n_{clust},n_{sub},se_{d},sp_{d})$$ (Eq 2)

$$\varepsilon_{undertreat}=P(X_{i+}<c|\pi_{i}\geq T,\rho_{i},n_{clust},n_{sub},se_{d},sp_{d})$$ (Eq 3)

To be able to calibrate a value of the decision cut-off *c*, we need to choose what risk of over-and undertreatment (*ε*_*overtreat*_ and *ε*_*undertreat*_) we find acceptable for a point *π*_*i*_<*T* and another point *π*_*i*_≥*T*. Together, these two true prevalence points, which correspond with *LL* and *UL* of the grey zone, dictate the slope of the sigmoid curve illustrated in **Fig 2B** (the higher the desired steepness, the lower risk we are willing to accept). We express this as *ε*_*overtreat*_(*π*_*i*_ = *LL*)≤*E*_*overtreat*_ and *ε*_*undertreat*_(*π*_*i*_ = *UL*)≤*E*_*undertreat*_.

### Simulation framework to determine the decision cut-off *c*

The determination of the decision cut-off *c* is straightforward in a 1-stage LQAS ignoring the spatial heterogeneity of infections across clusters [8], but there is no analytical solution for a 2-stage LQAS allowing for spatial heterogeneity [6]. We therefore applied a Monte Carlo (MC) simulation framework to determine the decision cut-off *c*. In brief, we first simulated the distribution of the total number of positive test results (*X*_*i*+_) based on 10,000 MC draws from the 2-stage beta-binomial model (**Eq (1)**). A set of MC draws was produced for each combination of chosen prevalence threshold *T* and values of the limits of the grey zone (*LL*, *UL*), conditional on survey design (*n*_*clust*_, *n*_*sub*_) and spatial variation in true prevalences (*ρ*_*i*_).

Subsequently, for each combination of *c* ∈(0, *n*_*clust*_∙*n*_*sub*_) and prevalence threshold *T*, we calculated the probability of overtreatment *ε*_*overtreat*_ (**Eq (2)**) when *π*_*i*_ = *LL* and the probability of undertreatment *ε*_*undertreat*_ (**Eq (3)**) when *π*_*i*_ = *UL*. Suitable values of the decision cut-off *c* were those that resulted in both *ε*_*overtreat*_(*π*_*i*_ = *LL*)≤*E*_*overtreat*_ and *ε*_*undertreat*_(*π*_*i*_ = *UL*)≤*E*_*undertreat*_. When a multiple values of *c* satisfied the grey zone conditions (**Eq(2)** and (**Eq(3)**), we selected the value of decision cut-off *c* that offers the lowest attainable risk of *ε*_*undertreat*_ as we considered this the most important risk to mitigate.

**Fig 3:**
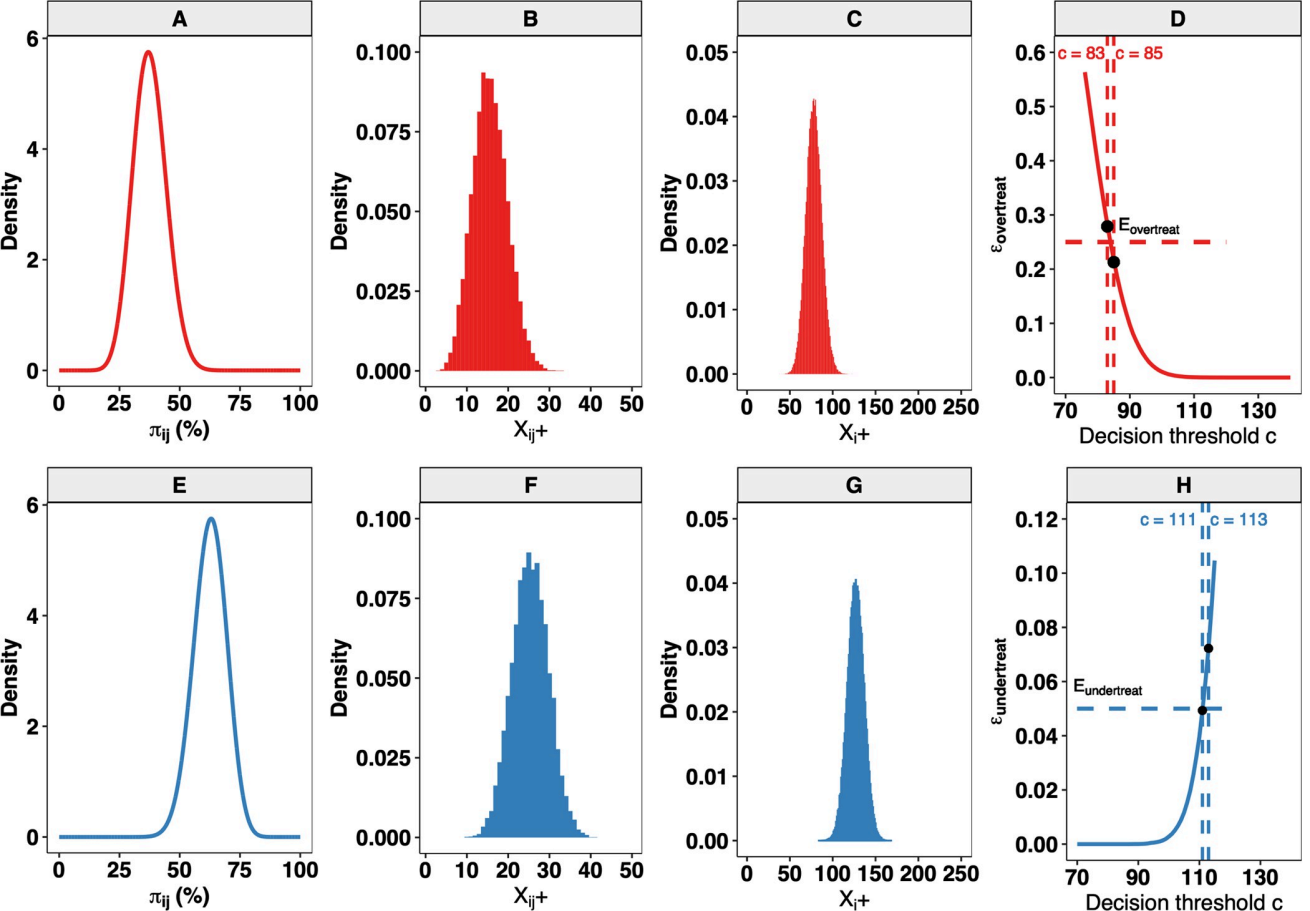
Determination of the decision cut-off *c* based on 2-stage LQAS framework allowing for imperfect tests. This figure describes the simulation framework to determine the decision cut-off ***c*** that allows for adequate (***E***_***overtreat***_ = **25**% and ***E***_***undertreat***_ = **5**%) decision-making at a true underlying prevalence ***π***_***i***_ at the implementation unit ***i*** equal to 37.5% (***LL***) and 62.5% (***UL***) when an imperfect theoretical diagnostic test *D* (***se***_***d***_ = **80**% and ***sp***_***d***_ = **98**%) was deployed to screen ***n***_***clust***_ = ***5*** and ***n***_***sub***_ = **50** subjects per cluster. We fixed the intra-cluster correlation ***ρ***_***i***_ = **0.02** at both limits of the grey zone. The bottom row graphs (**Panels A–D**) illustrate the process for the lower limit of the grey zone, whereas the bottom row graphs (**Panels E–H**) show this for the *UL* of the grey zone. The horizontal dotted line in **Panel D** represents the ***E***_***overtreat***_, wherein **Panel H** represents ***E***_***undertreat***_. The dotted vertical lines in these panels represent the decision cut-off ***c*** and the bullet the ***ε***_***overtreat***_ (**Panel D**) and ***ε***_***undertreat***_ (**Panel H**). All graphs are based on the same set of 10,000 Monte Carlo simulations.

In **Fig 3,** we further illustrate this process using the toy example of **Fig 2**. The top row (**Fig 3A–3D**) shows the process for the lower limit of the grey zone, whereas the bottom row (**Fig 3E–3H**) illustrates this for the upper limit of the grey zone. The first three panels in each row (**Fig 3A–3C** and **Fig 3E–3G**) represent the iterative process to determine the distribution of *X*_*i*+_ based on a 2-stage beta-binomial model; the fourth panel in each row (**Fig 3D** and **3H**) describes *ε*_*overtreat*_ and *ε*_*undertreat*_ across all potential values of *c* between zero and *n*_*clust*_∙*n*_*sub*_. Clearly, a decision cut-off *c* between 85 (**Fig 3D**: *ε*_*overtreat*_(*π*_*i*_ = *LL*)≤*E*_*overtreat*_) and 111 (**Fig 3H**: *ε*_*undertreat*_(*π*_*i*_ = *UL*)≤*E*_*undertreat*_) allowed for an acceptable risk of erroneous decision-making (see also **Fig 2B)**. Note that in this example, there are multiple options for *c*, but this may not always be the case. Indeed, it is anticipated that for some combinations of *n*_*clust*_, *n*_*sub*_, *se*_*d*_, *sp*_*d*_, *LL*, *UL*, and *ρ*_*i*_, there will not be any decision cut-off that fulfils the conditions *ε*_*overtreat*_(*π*_*i*_ = *LL*)≤*E*_*overtreat*_ and *ε*_*undertreat*_(*π*_*i*_ = *UL*)≤*E*_*undertreat*_.

### Application of the 2-stage LQAS framework to soil-transmitted helminths

#### Monitoring and evaluation of soil-transmitted helminth control programs as a case study

STH are a group of intestinal roundworms, including *Ascaris lumbricoides* (giant roundworm), *Trichuris trichiura* (whipworm), *Ancylostoma duodenale* and *Necator americanus* (hookworms). WHO recommends to control the STH-attributable morbidity through periodical administration of anthelmintic drugs to both children and other at-risk populations living in endemic areas. The frequency of these large-scale deworming programs is based on the observed prevalence of STH infections (any species). That is, at the start of the program, it is recommended to distribute drugs twice a year when the prevalence is at least 50% and once a year when the prevalence is at least 20%. During the implementation phase, the prevalence of any STH infection is periodically re-evaluated to verify whether objectives are being met, and if necessary, adjusting the frequency of drug administration (prevalence ≥50%: 3x PC / year; 50%> prevalence ≥20%: maintain PC frequency; 20%> prevalence ≥10%: 1x PC / year; 10%> prevalence ≥2%: 1x PC / 2 years; prevalence <2%: stop PC). To monitor and evaluate STH control programs, WHO recommends screening 5 schools and 50 children per school. Traditionally, STHs have been diagnosed by detecting worm eggs in a stool smear using a compound light microscope (the Kato-Katz thick smear method). This method is cheap and straightforward, yet notoriously imperfect, and sensitivity is more of a limitation (mainly for detection of low-intensity infections) than specificity [13,14]. Notably, there is a lack of studies investigating whether its performance is sufficient to make reliable program decision to stop PC and how use of better diagnostics or sampling strategies could lead to improvement.

We will illustrate the application of the 2-stage LQAS framework in the context of M&E of STH control programs, with the aims (*i*) to determine the required diagnostic performance when the current WHO survey design is applied, (*ii*) to further optimize the survey design for imperfect diagnostic tests, and (*iii*) to customize the sample throughput and cost per test according to the improvements in diagnostic performance. For each of these objectives, we applied the aforementioned 2-stage LQAS framework on strategically selected scenarios of *n*_*clust*_, *n*_*sub*_, *se*_*d*_, *sp*_*d*_, *LL*, *UL*, and *ρ*_*i*_, and verified whether we could render a decision cut-off *c* that fulfils the conditions *ε*_*overtreat*_(*π*_*i*_ = *LL*)≤*E*_*overtreat*_ and *ε*_*undertreat*_(*π*_*i*_ = *UL*)≤*E*_*undertreat*_. To allow for two different operational definitions of ‘reliable’ program decision-making (‘adequate’ *vs*. ‘ideal’), we set the *E*_*undertreat*_ at 5% for both definitions, whereas we set *E*_*overtreat*_ at either 25% (‘adequate’) or 10% (‘ideal’). These values for *E*_*undertreat*_ and *E*_*overtreat*_ have also been previously used to determine the sensitivity and specificity for diagnostic tests for other helminth diseases [5,6]. **S1 Table** provides an overview of the different *n*_*clust*_, *n*_*sub*_, *se*_*d*_, *sp*_*d*_, *LL*, *UL*, *ρ*_*i*_, *E*_*undertreat*_ and *E*_*overtreat*_ that were considered in our simulations and **S2 Table** shows a summary of the simulated diagnostic methods. In the following sections, we will briefly justify the choices made to meet the objectives. Also note that we assume that sensitivity and specificity of a diagnostic test *D* do not vary across individuals, even though this assumption does not really hold, as the sensitivity of detecting worm eggs in stool depends on the intensity of STH infection, which is known to vary between individuals [15].

#### Determine the required diagnostic performance when applying the current WHO survey design

To determine the required diagnostic performance when applying the current WHO survey design (*n*_*clust*_ = 5 and *n*_*sub*_ = 50) across each of the 4 program decision thresholds *T* (2%, 10%, 20% and 50%), we varied both *se*_*d*_ and *sp*_*d*_ from 60% to 100% (with 1% increments), resulting in a 41 × 41 grid of hypothetical diagnostic tests.

Given the wide range in program decision thresholds *T* (2% to 50%), we opted to define the limits of the grey zone proportional to *T*. We arbitrarily set the limits at *T*±25%. For example, for a *T* equal to 2%, *LL* and *UL* were set at 1.5% and 2.5%, respectively, while for a *T* equal to 50%, these numbers were 37.5% and 62.5%. We assumed that spatial heterogeneity (*ρ*_*i*_ = 0.02) was the same for all true prevalences of infection *π*_*i*_. For example, this assumption translates to a central 95%-confidence interval (95%-*CI*) of cluster-level true prevalence *π*_*ij*_ of 36.2–63.8% for an implementation unit *i* with an expected cluster-level true prevalence *π*_*i*_ of 50%; analogously, for *π*_*i*_ = 2% this translates to a 95% *CI* of 0.0–7.3%.

#### Optimise the survey design when using an imperfect diagnostic test

To optimise the survey design, we explored the impact of the width of the grey zone (*LL* and *UL*), *se*_*d*_, *sp*_*d*_, *n*_*clust*_ and *ρ*_*i*_ on *n*_*sub*_. To this end, we determined the required *n*_*sub*_ that allowed for adequate (*E*_*undertreat*_ = 5% and *E*_*overtreat*_ = 25%) program decisions around a program decision threshold of 2% for different scenarios of the width of the grey zone (*T*±25%, ±40%, ±50%, ±60% and ±75%), *se*_*d*_ (80%−100%, with 1% increments), *sp*_*d*_ (98%−100%, with 0.1% increments), *n*_*clust*_ (5, 10, 15 and 20) with *p*_*i*_ (initially) fixed at 0.02. We focused on *T* = 2% because the requirements for *n*_*clust*_ and *n*_*sub*_ become more stringent as a function with decreasing values of *T* [6].

Finally, we further assessed the impact of the geographical variation in prevalence between clusters (*ρ*_*i*_) and the number of sampled clusters (*n*_*clust*_) on the minimum required number of subjects per cluster (*n*_*sub*_). To this end, we arbitrarily fixed the limits of the grey zone at 1% and 3%, assumed a theoretical diagnostic test with *se*_*d*_ = 80% and *sp*_*d*_ = 98%, explored three values for *n*_*clust*_ (10, 15 and 20) and varied the intra-cluster correlation *ρ*_*i*_ from 0.012 (lower spatial heterogeneity of infections across the clusters) to 0.032 (higher spatial heterogeneity of infections across the clusters) with 0.004 increments.

We also estimated how the total survey costs (*C*_*tot*_) may change with the diagnostic test characteristics (*se*_*d*_ and *sp*_*d*_), which dictate the minimum required *n*_*clust*_ and *n*_*sub*_, given (*LL*, *LL*, *E*_*undertreat*_ and *E*_*overtreat*_), reagent costs, and sample throughput. As a benchmark, we used a theoretical reference diagnostic test *D*_*t*1_ (*se*_*dt*1_ = 80% and *sp*_*dt*1_ = 98%, as in the example used in **Fig 2**) for which we assume that reagent costs and throughput are the same as a single Kato-Katz thick smear, as recently estimated by Coffeng et al.[16]. These cost estimates include (*i*) the reagent cost for collection (0.57 *US*$ per sample) and testing (1.38 *US*$ per sample), (*ii*) the salary for a single mobile field team comprised of one nurse and three laboratory technicians (90 *US*$ per day, assuming 8 working hours), and (*iii*) the cost per day for car rental, including salary of the driver and gasoline (90 *US*$ per day). As in Coffeng et al. [16], we adopt the assumption that a team collects samples in the morning (8:00–12:00) and that all collected samples are processed in the afternoon (13:00–17:00), which implies that the number of samples that can be collected daily is limited. Therefore, we calculated for each survey design the number of working days (*n*_*days*_) required to screen all recruited subjects. For this, as by Coffeng et al.[16], we assume that it takes a person on average 412 seconds to process and test a single stool sample (which in Coffeng et al.’s analysis included preparation and examination of stool smear, digitization of demographic information, and recording the egg counting results). The sample throughput for the hypothetical reference test was 9 samples per hour, whereas the reagent cost to collect samples and the reagent cost to test samples were 0.57 *US*$ and 1.38 *US*$ respectively.

The equation to calculate *n*_*days*_ is described below:

$$n_{days}=n_{clust}∙n_{sub}/(3\;lab\;technicians∙4\;hours∙sample\;throughput)$$ (Eq 4)

Finally, (*iv*) we included a cost of 180 *US*$ (salary for one team + one car rental) per school included in the survey. This cost also covers the time required to inform the schools of the purpose of the study. The total survey cost *C*_*tot*_ is as follows:

$$\begin{array}{l} C_{\mathrm{tot}}=\mathrm{cost}\;\mathrm{of}\;\mathrm{consumables}\;\mathrm{to}\;\mathrm{collect}\;\mathrm{and}\;\mathrm{process}\;\mathrm{samples}\\ +\mathrm{operational}\;\mathrm{cost}\;\mathrm{to}\;\mathrm{collect}\;\mathrm{and}\;\mathrm{process}\;\mathrm{samples}\\ +\mathrm{cost}\;\mathrm{to}\;\mathrm{inform}\;\mathrm{school}\\ =n_{\mathrm{clust}}\cdot n_{\mathrm{sub}}\cdot (\mathrm{cost}\;\mathrm{to}\;\mathrm{collect}\;\mathrm{sample}+\mathrm{reagent}\;\mathrm{cost}\;\mathrm{to}\;\mathrm{test}\;\mathrm{sample})\\ +n_{\mathrm{days}}\cdot 180\;US\$\\ +n_{\mathrm{clust}}\cdot 180\;US\$. \end{array}$$ (Eq 5)

We subsequently applied *C*_*tot*_ to determine which *n*_*clust*_ *and n*_*sub*_ represent the number of clusters and subjects per cluster that allow for adequate (*E*_*undertreat*_ = 5% and *E*_*overtreat*_ = 25%) program decisions around a program decision threshold *T* of 2% (*LL* = 1% and *UL* = 3%) for a given *se*_*d*_ and *sp*_*d*_. We fixed *ρ*_*i*_ at 0.02. We assumed that (*i*) there were no additional costs for the laboratory infrastructure, (*ii*) that the team only received compensation during working days (Monday–Friday), and (*iii*) did not take any breaks during processing.

#### Customize the sample throughput and cost per test according to the improvements in diagnostic performance

To assess how the total cost of a survey depends on the diagnostic test performance (which dictates the minimal adequate design in terms of *n*_*clust*_ and *n*_*sub*_), the sample throughput and cost per test, we defined three theoretical diagnostic methods *D*_*t*1_−*D*_*t*3_ with the same sensitivity ($se_{dt1}=se_{dt2}=se_{dt3}=80\%$) but varying specificity ($sp_{dt1}=98\%,\;sp_{dt2}=96\%$ and *sp*_*dt*3_ = 94%) and apply these to varying scenarios of sample throughput (3–50 samples processed by one person in an hour, with 1 sample increment) and cost per test (1 *US*$−10 *US*$, with 0.01 *US*$ increment) applying **Eqs (4)** and **(5)** to estimate the total survey cost. For each combination of the parameters mentioned above, we then expressed the entire survey cost relative to the total cost of a survey based on the theoretical reference diagnostic test *D*_*t*3_ (*se*_*dt*3_ = 80% and *sp*_*dt*3_ = 94%) with cost characteristics as for single Kato-Katz thick smears. In each of the scenarios, *ρ*_*i*_ was fixed at 0.02, and the grey zone was defined as *T*±50% (*LL* = 1% and *UL* = 3%).

#### Application of the framework to develop evidence-based guidelines and direct R&D

To further illustrate how our framework can contribute to the development of more evidence-based WHO guidelines, we explored the required number of subjects per cluster when 10, 15, and 20 clusters (intra-cluster correlation *ρ*_*i*_ of 0.02) were sampled that allows for adequate program decision-making (*E*_*overtreat*_ = 25% and *E*_*undertreat*_ = 5%). We considered the 2% prevalence threshold (LL = 1% and UL = 3%) separately for two theoretical diagnostic tests *D*_*tpp*1_ ($Se_{tpp1}=60\%$ and $Sp_{tpp1}=99\%$) and *D*_*tpp*2_ ($Se_{tpp2}=86\%$ and $Sp_{tpp2}=94\%$). The values for the diagnostic performance of both tests were based on the minimal required sensitivity and specificity defined in the recently published WHO target product profiles (TPPs) for M&E of STH control programs [17]. We further estimated the total survey cost using equations **Eq (4)** and **Eq (5).** To this end, we used the required sample throughput (minimal: 7 samples per person per hour; ideal: 10 samples per person per hour) and reagent costs (minimal: 3 *US*$, ideal: 1 *US*$) listed in the WHO TPPs [17].

Further, we used our framework to gain insights into where R&D should be directed. For this, we fixed the diagnostic performance for single Kato-Katz thick smear *KK* (*se*_*kk*_ = 55% and *sp*_*kk*_ = 95%) to detect low intensity infections [18–21]. Then, we considered two hypothetical improved Kato-Katz thick smear methods, one with improved sensitivity *KK*_*se*_(*se*_*kkse*_ = 60% and *sp*_*kkse*_ = 95%) and one with improved specificity *KK*_*sp*_ (*se*_*kksp*_ = 55% and *sp*_*kksp*_ = 99%). We then estimated the required number of subjects per cluster and the associated total survey cost when 10, 15, and 20 clusters were sampled around the 2% program prevalence threshold (LL = 1% and UL = 3%) that allow for adequate decision-making (*E*_*overtreat*_ = 25% and *E*_*undertreat*_ = 5%). Additionally, we estimated the highest possible reagent cost per test for the diagnostic test *KK*_*sp*_. For this, we determined the survey cost for this improved Kato-Katz thick smear with varying scenarios of sample throughput (3–50 samples) and reagent cost to test one sample (1 *US*$−10 *US*$) relative to the total cost of a survey based on the hypothetical diagnostic test that meets the WHO TPPs for M&E of STH control program in terms of diagnostic performance *D*_*tpp*1_ (*Se*_*tpp*1_ = 60% and*Sp*_*tpp*1_ = 99%), sample throughput (7 samples per person per hour) and reagent cost (3 *US*$ per test), while setting the number of clusters at 10.

## Results

### The required diagnostic performance when applying the current WHO survey design

**Fig 4:**
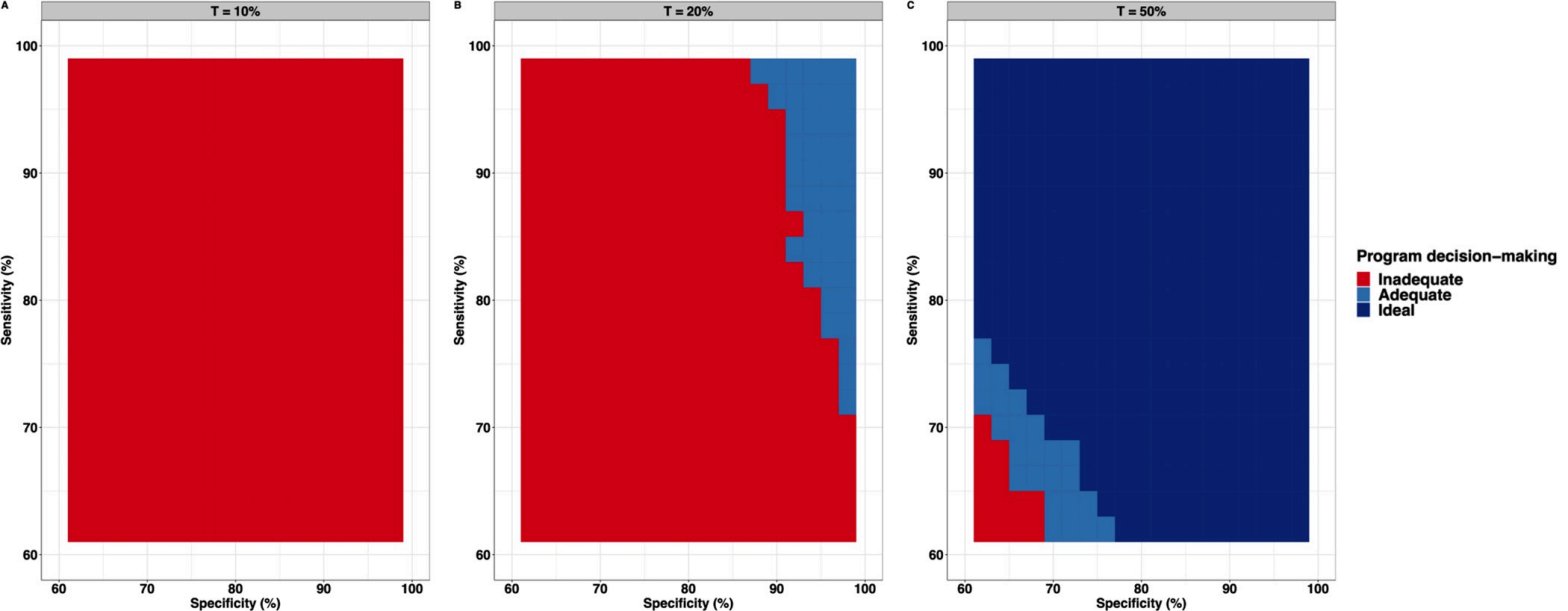
The required sensitivity and specificity when applying the current WHO study design. This figure indicates the required sensitivity and specificity when applying the current WHO study design (***n***_***clust***_ = **5** and ***n***_***sub***_ = **50**) that allow for adequate ($E_{overtreat}=25\%\;and\;E_{undertreat}=5\%)$ or ideal ($E_{overtreat}=10\%\;and\;E_{undertreat}=5\%)$ decision-making around each of the four program prevalence thresholds *T* (**Panel A**: 10%; **Panel B**: 20%; **Panel C**: 50%). The intra-cluster correlation ***ρ***_***i***_ was fixed at 0.02, and the limits of the grey zone were defined as a proportion of the program prevalence threshold T(LL=T−0.25∙T;UL=T+0.25∙T). The red area indicates that the combination of the survey design (***n***_***clust***_, ***n***_***sub***_) and the test performance (***se***_***d***_ and ***sp*_*d*_**) was inadequate for decision-making, while the light blue and the dark blue represent combinations that allow for adequate ($E_{overtreat}=25\%\;and\;E_{undertreat}=5\%)$ and ideal $\left(E_{overtreat}=10\%\;and\;E_{undertreat}=5\%\right)$ decision-making, respectively. All graphs are based on the same set of 10,000 Monte Carlo simulations.

**Fig 4** illustrates the required sensitivity and specificity when applying the WHO survey design (*n*_*clust*_ = 5 and *n*_*sub*_ = 50 per cluster) around the three highest program prevalence thresholds *T* (**Fig 4A**: 10%; **Fig 4B**: 20%; **Fig 4C**: 50%). We fixed the intra-cluster correlation *ρ*_*i*_ at 0.02 and defined the limits of the grey zone as a proportion of the *T* (*LL* = *T*−0.25∙*T*; *UL* = *T*+0.25∙*T*). Each of the panels represents a contour plot highlighting all possible combinations of sensitivity and specificity that rendered a decision cut-off *c* that fulfils $\varepsilon_{overtreat}(\pi_{i}=LL)\leq E_{overtreat}$ and $\varepsilon_{undertreat}(\pi_{i}=UL)\leq E_{undertreat}$ for both adequate (all combinations within the light blue and dark blue area) and ideal program decision-making (all combinations in the dark blue area). The red area indicates all combinations of sensitivity and specificity that did not render a decision cut-off *c* that fulfilled the conditions for adequate decision-making. Three crucial aspects can be observed in **Fig 4**. First, the requirements for diagnostic test performance become less stringent when the program threshold is closer to 50%. None of the combinations of sensitivity and specificity allowed for adequate or ideal decision-making around program thresholds of 10% (areas of **Fig 4A** is entirely red) and 2% (not shown as it is identical to 10%). For thresholds of 20% and 50%, program decisions were either adequate (**Fig 4B** contains red and light blue) or adequate to ideal (**Fig 4C** contains all three colours), respectively. Second, the requirements for specificity and sensitivity are inversely correlated. For instance, when adequate performance program decisions are made around a 50% threshold (**Fig 4C**) and with a diagnostic test with specificity equal to 60%, the sensitivity must be at least 70% and vice versa. Third, the requirements for specificity are more stringent than those for sensitivity, which became already apparent in the scenario where program decision-making is made around a *T* of 20% (**Fig 4B**). When employing a diagnostic method with a perfect specificity, the sensitivity should not drop below 72% to guarantee adequate decision-making. In contrast, the specificity can only drop to 86% when the sensitivity is set at 100%.

### Optimise the survey design when using an imperfect diagnostic test

**Fig 5:**
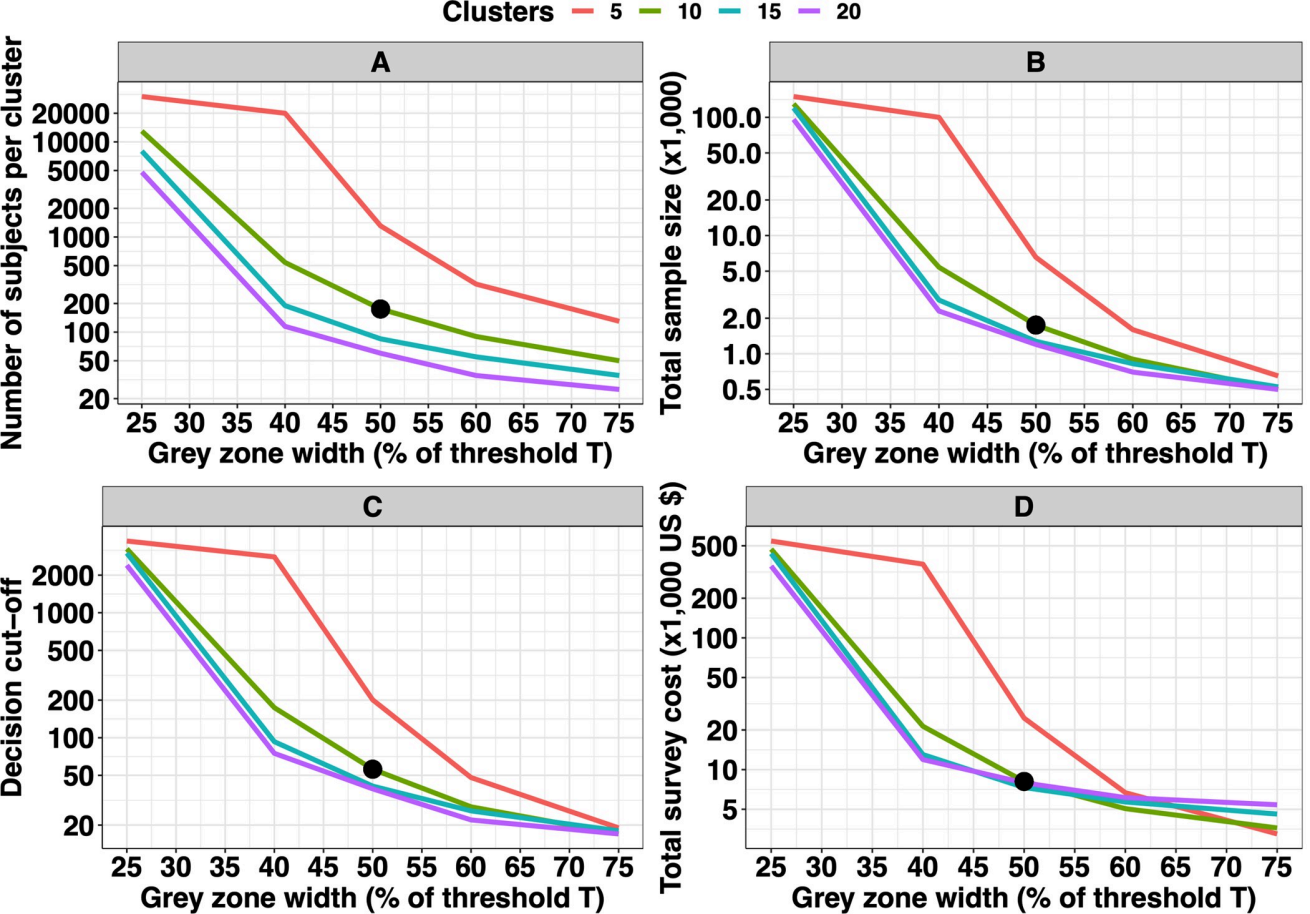
Impact of the width of the grey zone on program decision-making when applying imperfect diagnostics. This figure illustrates the impact of the width of the grey zone on the number of subjects per cluster (**Panel A**), the total number of subjects sampled across ***n***_***clust***_ clusters on the minimum required survey design for adequate program decision-making (***E***_***overtreat***_ = **25**% and ***E***_***undertreat***_ = **5**%) (**Panel B**), the decision threshold ***c* (Panel C),** and the associated total survey cost (**Panel D**). We considered program prevalence threshold ***T*** of 2%, fixed the intra-cluster correlation ***ρ***_***i***_ at 0.02 and assumed a theoretical diagnostic test *D*_*t*1_ (***se***_***dt*1**_ = **80**% and ***sp***_***dt*1**_ = **98**%). The width of the grey zone is expressed as a proportion of program prevalence threshold ***T***. The black bullet across the four panels indicates the chosen hypothetical reference grey zone width (***T***±**50**%), which was chosen to further illustrate the impact of the diagnostic performance (**Fig 6**) and geographical variation in prevalence between clusters on program decision-making (**Fig 7**), and the relative change in total survey cost based on newer diagnostic tests (**Fig 8**). All graphs are based on the same set of 10,000 Monte Carlo simulations.

We now proceed to optimise the survey design for a program prevalence threshold of 2%, for which the WHO-recommended survey design is not sufficient. The first step towards this is to evaluate how stringency (i.e., grey zone width) affects the minimum survey design. **Fig 5** illustrates the impact of the width of the grey zone on the number of subjects per cluster (*n*_*sub*_; **Fig 5A**), the total number of subjects sampled across *n*_*clust*_ clusters (*n*_*tot*_; **Fig 5B**), the corresponding decision cut-off (*c*; **Fig 5C**), and the associated survey costs (*C*_*tot*_; **Fig 5D**) required for adequate program decision-making $\left(E_{overtreat}=25\%,\;E_{undertreat}=5\%\right)$. We fixed the intra-cluster correlation *ρ*_*i*_ at 0.02 and assumed the use of a theoretical diagnostic test *D*_*t*1_ (*se*_*dt*1_ = 80% and *sp*_*dt*1_ = 98%). Each of these four metrics (*n*_*sub*_, *n*_*tot*_, *c* and *C*_*tot*_) decreased as the width of the grey zone became wider (i.e., when less stringent requirements for decision-making are defined). For example, when the limits of the grey zone were defined as 40% of the program threshold (LL=T−0.4∙T=0.02−0.02∙0.40=0.012;UL=T+0.4∙T=0.02+0.02∙0.40=0.028) and 10 clusters were randomly selected, the minimum required number of subjects per cluster was 540 (*n*_*tot*_ = 5,400), where this was 175 (*n*_*tot*_ = 1,750) when the limits were defined as *T*±50% (*LL* = 1%, *UL* = 3%) (**Fig 5A** and **B**). Additionally, it is important to note that this difference in *n*_*tot*_ subjects further decreased when the width of the grey zone became wider. Indeed, when the width of the grey zone was *T*±75%, the required *n*_*tot*_ subjects was 650 when *n*_*clust*_ equalled 5 and 500 when *n*_*clust*_ was set at 10 (**Fig 5B**). Furthermore, **Fig 5C** shows very similar trends as **Fig 5B** because the decision cut-off also decreased when the width of the grey zone became wider. Also, for relatively small grey zone, a higher number of clusters (either 15 or 20) is most cost-efficient. As a result, it was not unexpected to observe that for a width of the grey zone defined as *T*±75%, sampling 5 clusters became the most cost-efficient design (**Fig 5D**).

To facilitate interpretation of the impact of the other parameters on the program decision-making, we fixed the limits of the grey zone in all next figures to 1% (= *LL*) and 3% (= *UL*). These limits corresponded to *T*±50% (black dots in **Fig 5**) and resulted in a total sample size *n*_*tot*_ of about 2,000 (200 subjects per cluster) and a decision cut-off of 60 when 10 clusters were sampled, a sample size that we considered feasible under field conditions.

**Fig 6:**
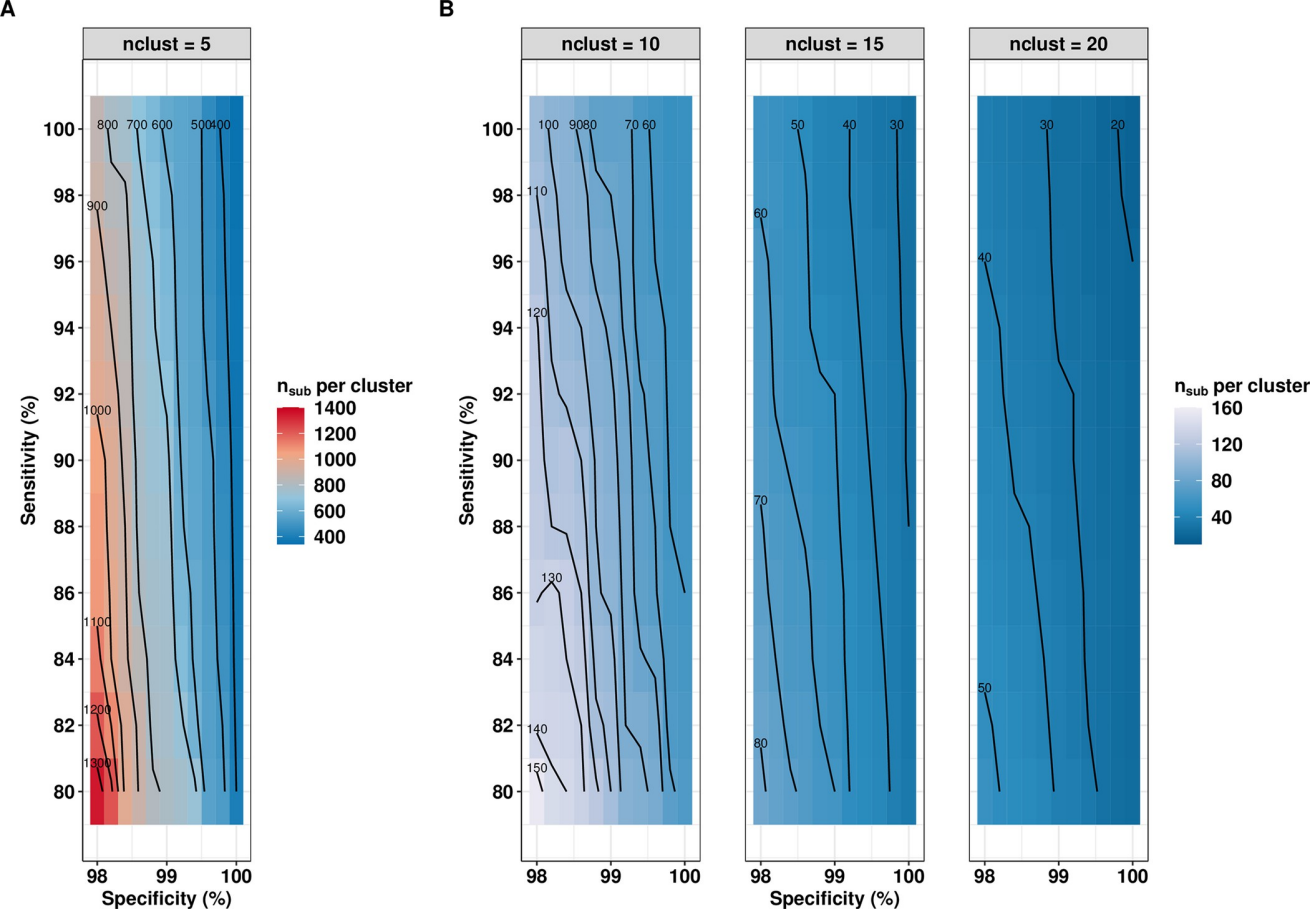
Impact of the diagnostic performance on program decision-making. The contour lines illustrate the relationship between specificity (x-axis), the sensitivity (y-axis), and the minimum number of subjects per cluster ***n***_***sub***_ (contour lines) for adequate program decision-making (***E***_***overtreat***_ = **25**% and ***E***_***undertreat***_ = **5**%) around a 2% program prevalence threshold for the number of clusters ***n***_***clust***_ equal to 5, 10, 15 and 20. We fixed the intra-cluster correlation ***ρ***_***i***_ at 0.02 and the limits of the grey zone at 1% and 3%. All graphs are based on the same set of 10,000 Monte Carlo simulations.

We now proceed to evaluate the impact of diagnostic test performance on the minimum survey design. **Fig 6** illustrates the impact of the diagnostic performance (*se*_*d*_≥80% and *sp*_*d*_≥98%) and the number of sampled clusters (*n*_*clust*_: 5, 10, 15 and 20) on the minimum required number of subjects per cluster (*n*_*sub*_) for adequate program decision-making (*E*_*overtreat*_ = 25% and *E*_*undertreat*_ = 5%) around a 2% program prevalence threshold. We fixed the intra-cluster correlation *ρ*_*i*_ at 0.02. Generally, this figure highlights three important aspects. First, improving the diagnostic performance can substantially further reduces the minimum required number of subjects per cluster and the total sample size. For example, when sampling 5 clusters and deploying a diagnostic test *D* (*se*_*d*_ = 80% and *sp*_*d*_ = 98%), the minimum required number of subjects per cluster was ~1,300. However, it dropped to 400 subjects per cluster when the diagnostic test specificity was set at 99.9% (**Fig 6A**). Second, these panels confirm that specificity is more important than sensitivity when evaluating prevalence around 2%. In other words, improving the specificity has more impact on the minimum required sample size than improving the sensitivity. This can be readily seen from the almost vertical orientation of the contour lines in all the panels of **Fig 6**, which indicate that sample size changes fastest with specificity (i.e., about perpendicular to the contour lines). Third, as expected, sampling fewer clusters leads to a greater number of subjects per cluster and ultimately to an increased total sample size. For example, when sensitivity was set at 80% and specificity was set at 98%, the minimum required number of subjects per cluster was ~1,300 when 5 clusters were sampled (*n*_*tot*_ = 6,500). For 10, 15 and 20 clusters, these numbers were about 150 (*n*_*tot*_ = 1,500), 80 (*n*_*tot*_ = 1,200), and 50 (*n*_*tot*_ = 1,000), respectively (**Fig 6B**).

**Fig 7:**
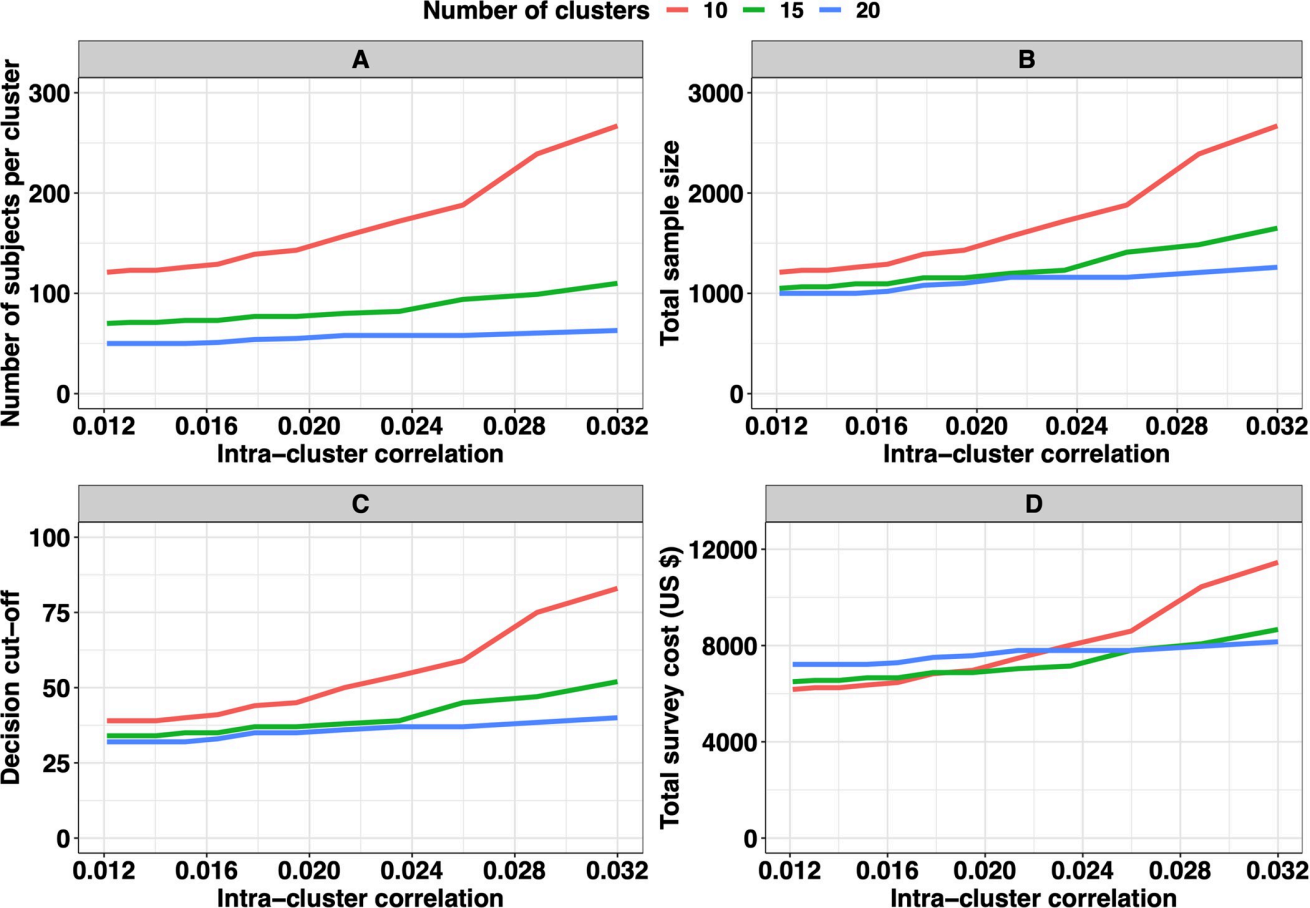
Impact of geographical variation in prevalence between clusters on program decision-making. This figure illustrates the impact of the geographical variation in prevalence (expressed as intra-cluster correlation, which ranged from 0.012 to 0.032) on the number of subjects per cluster (***n***_***sub***_; **Panel A**), the total number of subjects sampled across ***n***_***clust***_ (***n***_***tot***_; **Panel B**), the corresponding decision cut-off ***c*** (**Panel C**), and the associated total survey costs (***C***_***tot***_; **Panel D**) required for adequate program decision-making (***E***_***overtreat***_ = **25**% and ***E***_***undertreat***_ = **5**%) around a 2% program prevalence threshold. In this figure, the sensitivity and specificity were set at **80**% and **98**%, respectively. The limits of the grey zone at 1% and 3%. All graphs are based on the same set of 10,000 Monte Carlo simulations.

Last, we consider the impact of geographical variation on the optimal study design for a threshold of 2%, assuming test sensitivity and specificity of 80% and 98%, respectively. **Fig 7** displays the impact of the geographical variation in prevalence (expressed as intra-cluster correlation, which ranged from 0.012 to 0.032) on the number of subjects per cluster (*n*_*sub*_; **Fig 7A**), the total number of subjects sampled across *n*_*clust*_ (*n*_*tot*_; **Fig 7B**), the corresponding decision cut-off (*c*; **Fig 7C**), and the associated total survey costs (*C*_*tot*_; **Fig 7D**) required for adequate program decision-making (*E*_*overtreat*_ = 25% and *E*_*undertreat*_ = 5%) around a 2% program prevalence threshold. Each of these four metrics increased as a function of more heterogeneously distribution of STH infections. In other words, if the spatial heterogeneity is different between implementation units, the optimal design for decision-making will be different. For example, when fixing the number of clusters at 10, a minimum of 120 subjects per cluster are required when the intra-cluster correlation *ρ*_*i*_ equalled 0.012, while 188 subjects per cluster are needed when *ρ*_*i*_ was set at 0.025 (**Fig 7A**). Additionally, for the total survey cost (**Fig 7D**), sampling fewer clusters are preferred when infections are more homogeneously among clusters (*ρ*_*i*_<0.017). For example, it is apparent from **Fig 7D** that sampling 10 or 15 clusters was the most cost-effective when the intra-cluster correlation ranged from 0.012 to 0.017. However, in case of a more heterogeneous distribution, **Fig 7D** indicates that it becomes more cost-effective to sample more clusters and fewer subjects per cluster. For example, when fixing the intra-cluster correlation *ρ*_*i*_ at 0.012, the total survey cost was ~6,000 *US*$ when 10 clusters were sampled and ~7,000 *US*$ in case 20 clusters were sampled. In contrast, for an intra-cluster correlation of 0.025, these numbers were ~8,500 *US*$ for 10 clusters and ~7,8000 *US*$ for both 15 and 20 clusters (**Fig 7D**).

### Customize the sample throughput and cost per test according to the improvements in specificity

**Fig 8:**
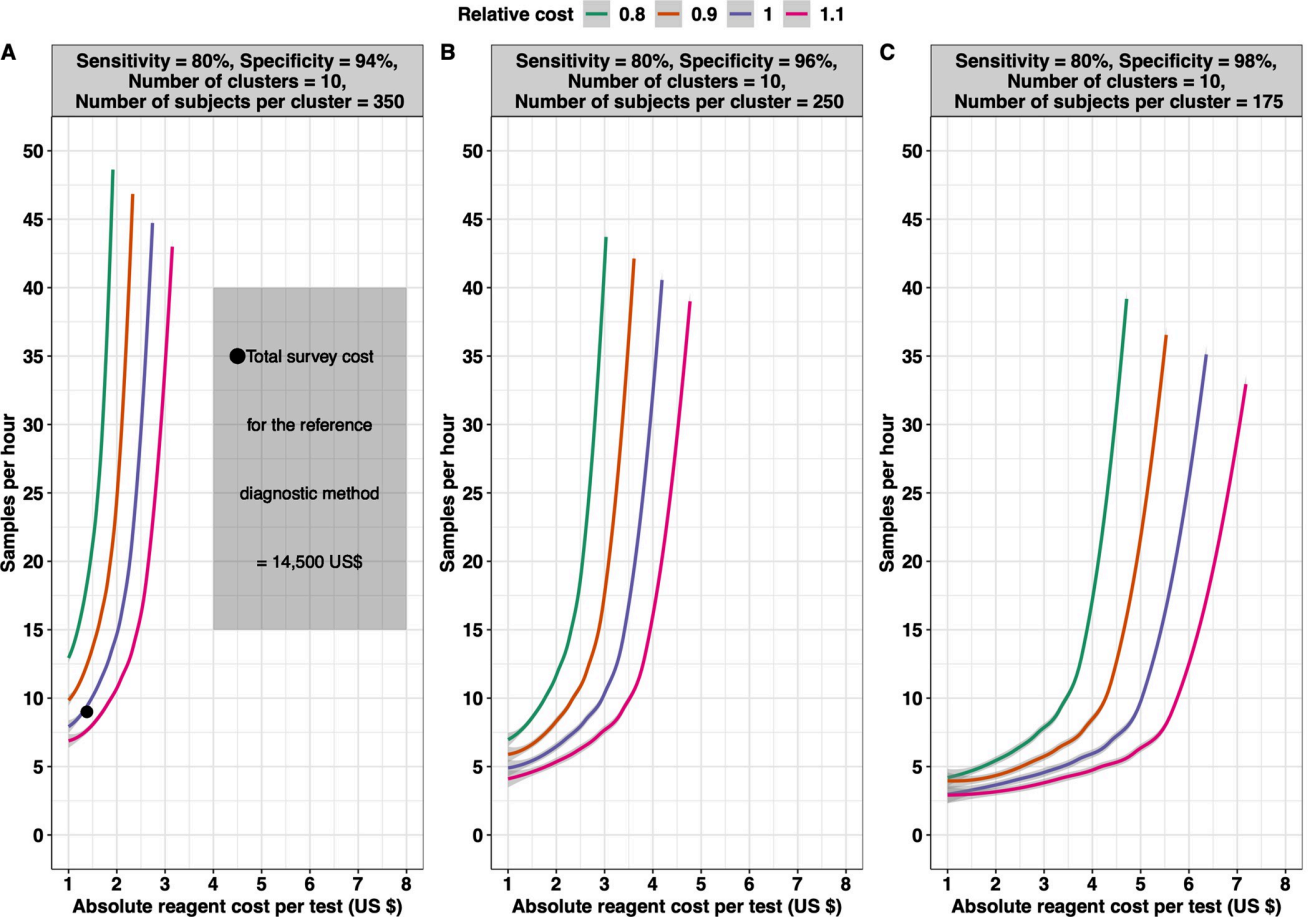
The relative total survey cost of imperfect diagnostic tests with varying sample throughput and reagent costs. The contour lines illustrate the total survey cost when applying imperfect diagnostic tests with varying sample throughput and reagent cost to test one sample relative to the total cost of a survey based on the hypothetical reference diagnostic test with the sample throughput (9 samples per hour per person) and cost characteristics (1.38 US$ reagent test cost) of single Kato-Katz thick smear. We considered three hypothetical diagnostic tests ***D***_***t*3**_ (reference diagnostic test; **Panel A**), ***D***_***t*2**_ (**Panel B**), and ***D***_***t*3**_ (**Panel C**), each with a different diagnostic performance (${D_{t1}}_{:\;}se_{dt1}=80\%,\;sp_{dt1}=98\%;\;D_{t2}:\;se_{dt2}=80\%,\;sp_{dt2}=96\%;\;D_{t3}:\;se_{dt3}=80\%,\;sp_{dt3}=94\%$). The intra-cluster correlation ***ρ***_***i***_ was set at **0.02** and the number of clusters at 10. The number of subjects per cluster and the total survey cost was defined as the minimum required number of subjects per cluster and minimal cost required for adequate decision-making (***E***_***overtreat***_ = **25**% and ***E***_***undertreat***_ = **5**%) around a program prevalence threshold of 2%, with the grey zone defined as ***T***±**50**% (***LL*** = **1**% and ***UL*** = **3**%). All three panels were based on 10,000 Monte Carlo simulations.

Let us now consider how the potentially increased cost and lower throughput of improved diagnostic tests may be offset by lower required sample sizes due to higher test specificity. **Fig 8** illustrates the survey cost for varying scenarios of sample throughput and reagent cost (i.e., the cost to test one sample) relative to the total cost of a survey based on the hypothetical reference diagnostic test with sample throughput (9 samples per hour per person) and cost characteristics (1.38 *US*$ reagent test cost) as single Kato-Katz thick smears. Three theoretical diagnostic methods *D*_*t*1_−*D*_*t*3_ were defined with varying specificity ($sp_{dt1}=98\%,\;sp_{dt2}=96\%$ and *sp*_*dt*3_ = 94%) while setting the sensitivity at 80% ($se_{dt1}=se_{dt2}=se_{dt3}=80\%$) for all three tests. As diagnostic test specificity will have an impact on the sample size (number of clusters and individuals per cluster) and thus the total survey costs (see **Fig 6**), we considered as reference the diagnostic method with the lowest specificity, *D*_*t*3_.

The estimated total survey cost for the reference diagnostic test *D*_*t*3_ (10 clusters and 350 individuals per cluster tested) was ~14,500 *US*$ (indicated by a black bullet in **Fig 6A**). From **Fig 8A**, it becomes clear that to keep the total survey cost the same (i.e., the purple line), the requirements for reagent costs and the sample throughput are correlated. For example, when we increase the reagent costs with 0.50 *US*$ *to* 1.88 *US*$, the required sample throughput should be at least 13 instead of 9 to result in the same total survey cost. The panel also indicates that the association describing the break-even point is non-linear. In other words, an increase of 0.50 *US*$ will not always require increasing the sample throughput by 4. Indeed, if we now further increase the reagent cost by 0.50 *US*$ *to* 2.38 *US*$, the required throughput needs to be increased up to 23 (increase of 10), underscoring that the compensation of increased reagent cost by the sample throughput is limited. In fact, for a survey based on the reference diagnostic test *D*_*t*3_, the reagent cost per test cannot exceed 2.90 *US*$.

**Fig 8B** and **C** illustrate the same trade-off between sample throughput and reagent costs for diagnostic tests *D*_*t*2_ (*se*_*dt*2_ = 80% and *sp*_*dt*2_ = 96%) and *D*_*t*1_ (*se*_*dt*1_ = 80%, *sp*_*dt*1_ = 98%), respectively. Generally, these panels indicate that when fixing the number of clusters at *n*_*clust*_ = 10, the improved diagnostic performance reduced the required number of subjects per cluster (350 *vs*. 250 *vs*. 175), which allowed for diagnostic tests with a lower sample throughput (downward shift of the contour lines) and higher reagent costs (shift of the contour lines to the right) while keeping the total survey cost the same (purple line). For example, to compensate for an 0.50 *US*$ increase in reagent costs, the required sample throughput should be at least 6 for diagnostic test *D*_*t*2_ and 4 for diagnostic test *D*_*t*1_ instead of 13 per hour. Furthermore, the highest possible reagent cost per test to ensure the same total survey cost for this improved diagnostic test was ~4.30 *US*$ for the diagnostic test *D*_*t*2_ and 6.50 *US*$ for diagnostic test *D*_*t*1_.

### Application of the framework to develop evidence-based guidelines and direct R&D

Given a 2% program prevalence threshold and a choice of grey zone width (*T*±50%) for adequate program decision-making (*E*_*overtreat*_ = 25% and *E*_*undertreat*_ = 5%), we investigated the potential contribution of our framework to more evidence-based WHO guidelines. For this, two hypothetical tests *D*_*tpp*1_ (*se*_*tpp*1_ = 60% and *sp*_*tpp*1_ = 99%) and *D*_*tpp*2_ (*se*_*tpp*2_ = 86% and *sp*_*tpp*2_ = 94%), both identified as potential diagnostic tests in the recently published by WHO TPPs for M&E of STH. The intra-cluster correlation (*ρ*_*i*_) was fixed at 0.02 when sampled 10, 15, and 20 clusters. Furthermore, we set the sample throughput (7 and 10 samples per person per hour) and reagent cost (1 *US*$, 3 *US*$ per test) [17]. **Table 3** illustrates the required number of subjects per cluster for each scenario. It is evident from this table that for a diagnostic test *D*_*tpp*1_ the number of subjects per cluster equalled 165 for 10 clusters, 92 for 15 clusters, and 60 for 20 clusters. For a diagnostic test *D*_*tpp*2_ the numbers were 330 (10 clusters), 162 (15 clusters), and 110 (20 clusters), respectively. Also, it becomes clear that the requirements for both sample throughput and reagent cost are less stringent for diagnostic test *D*_*tpp*1_. For approximately the same survey costs (~11,000 *US*$), the sample throughput and cost per test for diagnostic test *D*_*tpp*1_ can be 7 samples and 3 *US*$, respectively, while this needs to be 10 samples and 1 *US*$ for diagnostic test *D*_*tpp*2_.

**Table 3:** The use of the 2-stage LQAS framework to develop guidelines and strategic choices in R&D.

Test	*se*_*d*_ (%)	*sp*_*d*_ (%)	*n*_*clust*_	*n*_*sub*_	*n*_*tot*_	Sample throughput	Cost per test (US$)	*C*_*tot*_ (*US*$)
***Theoretical diagnostic method D meeting WHO TPPs***								
***D***_***tpp*1**_	60	99	10	165	1,650	10	3	10,165
							1	6,865
						7	3	11,226
							1	7,926
			15	92	1,380	10	3	9,696
							1	6,936
						7	3	10,583
							1	7,823
			20	60	1,200	10	3	9,684
							1	7,284
						7	3	10,455
							1	8,055
***D***_***tpp*2**_	86	94	10	330	3,300	10	3	18,531
							1	11,931
						7	3	20,652
							1	14,052
			15	162	2,430	10	3	15,020
							1	10,160
						7	3	16,582
							1	11,722
			20	110	2,200	10	3	14,754
							1	10,354
						7	3	16,168
							1	11,768
***Kato-Katz thick smear***								
***KK***	55	95	10	680	6,800	9	1.38	26,393
	55	95	15	340	5,100	9	1.38	21,145
	55	95	20	230	4,600	9	1.38	20,236
***Kato-Katz thick smear with improved sensitivity***								
***KK***_***se***_	60	95	10	540	5,400	10	3	29,178
							1	18,378
						7	3	32,649
							1	21,849
	60	95	15	280	4,200	10	3	23,994
							1	15,594
						7	3	26,694
							1	18,294
	60	95	20	190	3,800	10	3	22,866
						10 7	1 3	15,266 25,308
							1	17,708
***Kato-Katz thick smear with improved specificity***								
***KK***_***sp***_	55	99	10	200	2,000	10	3	11,940
							1	7,940
						7	3	13,225
							1	9,225
***KK***_***sp***_	55	99	15	110	1,650	10	3	11,065
							1	7,765
						7	3	12,126
							1	8,826
	55	99	20	80	1,600	10	3	11,712
							1	8,512
						7	3	12,740
							1	9,540

***se***_***d***_: sensitivity, ***sp***_***d***_: specificity, ***n***_***clust***_: number of clusters, ***n***_***sub***_: required number of subjects per cluster for each scenario, ***n***_***tot***_: total sample size, ***C***_***tot***_: total survey cost. This table illustrates the minimum required number of subjects per cluster and the total survey cost as the minimum cost required for the Kato Katz method used in low prevalence settings ***KK*** (***se***_***kk***_ = **55%** and ***sp***_***kk***_ = **95**%), an improved Kato Katz method (improving sensitivity by 5%, ***KK***_***se***_: ***se***_***kkse***_ = **60** and ***sp***_***kkse***_ = **95** or improving specificity by 4%, ***KK***_***sp***_: ***se***_***kksp***_ = **55**% and ***sp***_***kksp***_ = **99%**) for adequate program decision-making (***E***_***overtreat***_ = **25**% and ***E***_***undertreat***_ = **5**%) around a 2% program prevalence threshold. Likewise, the total survey cost and the number of subjects per cluster were estimated for the WHO recommended target product profiles (TPPs) for soil-transmitted helminth (STH) control programs (minimum: $se_{tpp2}=86\%,\;sp_{tpp2}=99\%,$ and ideal $se_{tpp1}=60\%,\;sp_{tpp1}=99\%)$ required. In this table, we fixed the intra-cluster correlation ***ρ***_***i***_ at **0.02** and the limits of the grey zone at **1**% and **3**%. The throughput **10** and **7**, as well as the cost per test of **1 *US***$ and **3 *US***$ was obtained from the WHO target throughput and target pricing per test for STH control programs, respectively. The number of clusters was set at 10, 15 and 20 clusters. A total of 10,000 Monte Carlo simulations was used.

**Fig 9:**
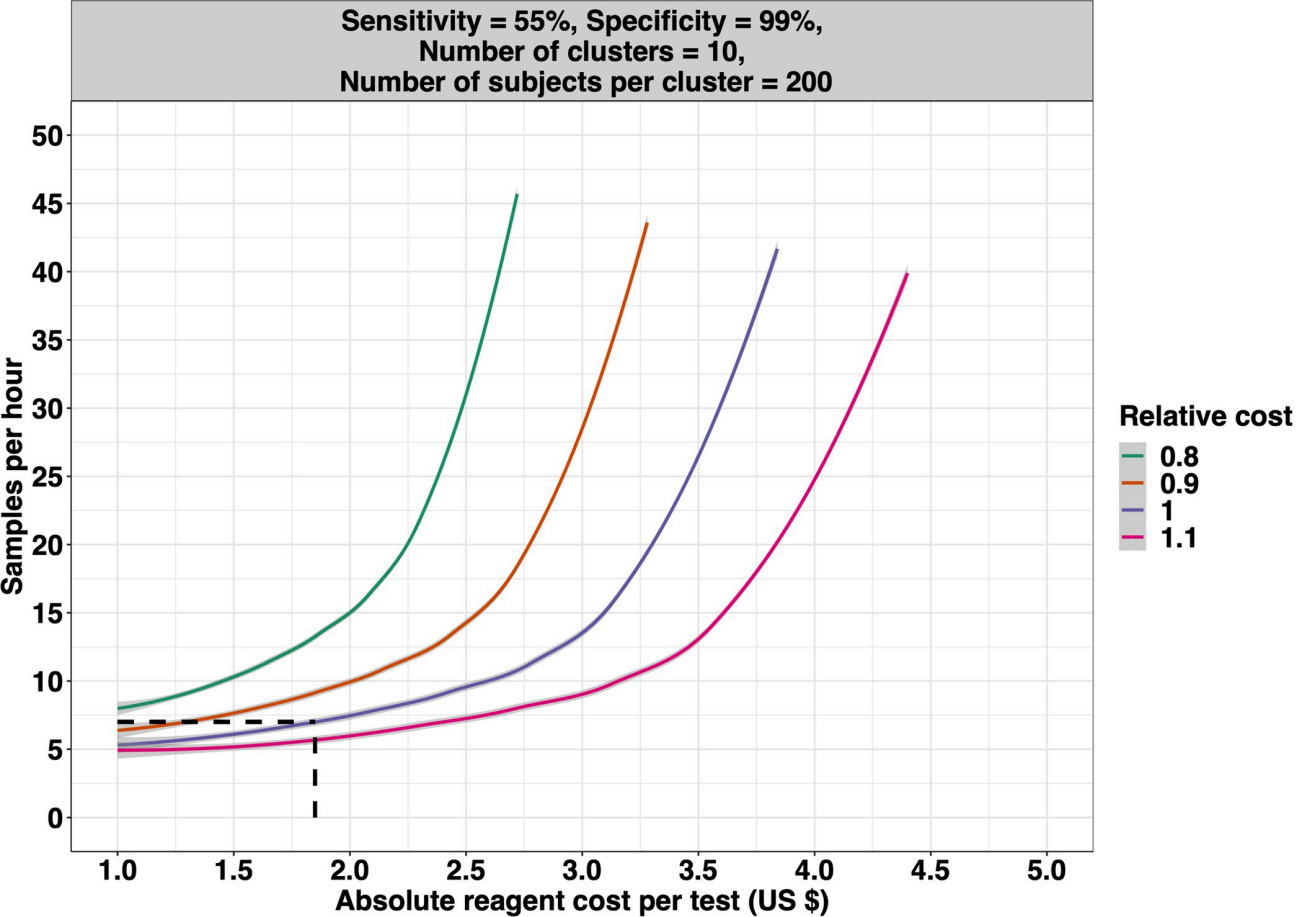
The relative total survey cost of Kato-Katz thick smear with improved specificity. The contour lines illustrate the total survey cost when applying a Kato-Katz thick smear with improved specificity ***KK***_***sp***_(***se***_***kksp***_ = **55**%, and ***sp***_***kksp***_ = **99**%) with varying sample throughput and reagent cost to test one sample relative to the total cost of a survey based on the soil-transmitted helminths target product profiles diagnostic performance ***D***_***tpp*1**_ (***se***_***tpp*1**_ = **60**% and ***sp***_***tpp*1**_ = **99**%) with sample throughput (7 samples per hour per person) and cost characteristics (**3 *US***$ reagent test cost). The dotted line indicates the maximum reagent cost per test for the improved Kato-Katz thick smear (**1.85 *US***$) when the sample throughput was set at 7. The intra-cluster correlation ***ρ***_***i***_ was set at **0.02** and the number of clusters at 10. The number of subjects per cluster was estimated as the required minimum number of subjects for adequate decision-making (***E***_***overtreat***_ = **25**% and ***E***_***undertreat***_ = **5**%) around a program prevalence threshold of 2%, with the grey zone defined as ***T***±**50**% (***LL*** = **1**%and ***UL*** = **3**%). The total survey cost was defined as the minimal cost required. The graph was based on 10,000 Monte Carlo simulations.

Let us now illustrate the potential contribution of our framework to R&D, considering a 2% program prevalence threshold (*LL* = 1% and *UL* = 3%). **Table 3** highlights that R&D should be directed towards improved specificity rather than sensitivity. For instance, if sampling 20 clusters with improved sensitivity, 190 subjects per cluster must be sampled, whereas only 80 are needed per cluster with improved specificity. Furthermore, we determined the highest possible reagent cost per test for the Kato-Katz thick smears with improved specificity *KK*_*sp*_ (*se*_*kksp*_ = 55% and *sp*_*kksp*_ = 99%). It can be observed from **Fig 9** that for the same total survey cost, the reagent cost per test for the Kato-Katz thick smear with improved specificity should not exceed 1.85 *US*$ when one person can only process 7 samples per hour. This cost per test could be relaxed to 2.40 *US*$ and 2.64 *US*$ when the sample throughput is increased to 9 and 10 samples, respectively.

## Discussion

Diagnostic tests currently deployed in M&E surveys are often imperfect. In the present study, we developed a 2-stage LQAS framework for program decision-making that allows for both imperfect diagnostics and spatial heterogeneity of infections. We applied the framework using M&E of STH control programs as a case study. For this, we explored the impact of the diagnostic performance, spatial heterogeneity and survey design on both the program decision-making around the prevalence decisions thresholds recommended by WHO and the total survey costs. In addition, we assessed the trade-off between cost per test, sample throughput and diagnostic performance of tests. Finally, we illustrated how our framework could support the development of evidence-based guidelines and direct R&D.

### Revision of WHO guidelines for M&E of STH programs may be warranted

The currently proposed survey design by WHO (5 schools and 50 subjects per school per implementation unit) allows for adequate (prevalence threshold of 20%) to ideal (prevalence threshold of 50%) program decision-making. However, the risk of an incorrect program decision around a prevalence threshold of 2% and 10% may be too high given the geographical heterogeneity (*ρ*_*i*_ = 0.02) in infection levels and predefined grey zone (*LL* = 1% and *UL* = 3%), as assumed here. Given that even perfect diagnostic tests cannot reduce this risk to an acceptable level under these assumptions (**S1 Fig**), revision of the WHO guidelines for M&E of STH programs may be warranted. To illustrate how our framework can contribute to the development of more evidence-based WHO guidelines, we provided an example calculation for the minimum survey design for diagnostic tests that satisfy the recently published WHO TPPs for M&E of STH control program. The resulting required sample size for both TPPs for M&E of STH was feasible under fields conditions (**Table 3**).

### A paradigm shift from sensitivity to specificity is warranted

Our study confirms that the requirements for diagnostic test parameters are inversely correlated [6] and that specificity rather than sensitivity is a critical diagnostic parameter to consider when NTD programs progress towards the end-game [5]. These observations have already been explained in more detail elsewhere [5,6], and hence we will restrict ourselves to underscoring their impact on research and development (R&D) of future diagnostic tests for NTD. First, we highlight that an important shift in paradigm on diagnostic performance will be required from the NTD research community. Where the focus has mainly been on the diagnostic sensitivity (sustained by the focus and conclusions of various research [22–24]), it has now become clear that obtaining a high diagnostic specificity will be the driving force for R&D. This need for more specific diagnostic tests is already apparent in the different TPPs that WHO recently published for onchocerciasis [25], lymphatic filariasis [26], soil-transmitted helminthiasis [17] and schistosomiasis [27]. In each of these TPPs, the required specificity did not drop below 94%, while the requirements for sensitivity were relaxed to 60%.

Second, we suggest a re-appraisal of the value of the current diagnostic standards. For example, a single Kato-Katz thick smear is the current diagnostic standard for STHs, and although it lacks sensitivity to detect infections of low intensity ([13,14,28,29]: *Ascaris*: ~55%, *Trichuris*: ~80%, hookworms: ~70%), it has a high specificity (≥98%) [19,30]. In other words, this cheap and simple method might still be valuable at the program’s endgame, though the survey design will need to be adapted accordingly. For example, assuming the aforementioned diagnostic performance of a single Kato-Katz thick smear and the operational cost for Kato-Katz thick smear described in the methods section, sampling 20 clusters and 230 subjects per cluster (total sample size = 4,600) is the most cost-effective survey design for *Ascaris* that allows for adequate program decision-making (*E*_*overtreat*_ = 25%, *E*_*undertreat*_ = 5%) around the 2% prevalence threshold (lower limit = 1% and upper limit = 3%) (**Table 3**). However, this survey design will require substantial financial resources (20,236 *US*$), though improvements to the Kato-Katz thick smear may drastically reduce this cost. Although there are several alternative diagnostics for STH (e.g., Mini-FLOTAC, McMaster, FECPAK^G2^ and qPCR), we strongly doubt whether they will outcompete the Kato-Katz thick smear. Their specificity too is not perfect [13,31] and they come with substantially more operational costs.

### Compensating improved diagnostics tests’ higher cost and lower throughput by lower sample size

Our results confirm that improving diagnostic performance results in smaller sample sizes for the same level of program decision-making. They also highlight that improving the diagnostic performance and the corresponding reduced sample sizes can compensate for more costly tests and lower sample throughput. In addition, our results highlight that there is a limit to the extent to which higher reagent costs can be compensated by lower sample throughput and vice versa. For example, in our theoretical scenarios, the highest possible cost per test equaled 6.50 *US*$ and the minimal sample throughput 3 samples per person per hour when test sensitivity is 80%, and specificity is 98%. These findings are crucial for R&D and will guide developers in making strategic choices. Indeed, different pairs of sensitivity and specificity have been included in the WHO TPP. Yet, each of them will come with a different survey design (see above), and because of this, requirements for both sample throughput and reagent cost per test will be different.

### Still a long way to go

Although we have illustrated the added value of our framework to develop more evidence-based M&E guidelines and make strategic R&D choices, there is still a long way to go. First, the optimal design will be specific to each implementation unit as it is expected that spatial heterogeneity will not be the same across implementation units due to local dispersion of houses and hygienic behaviour. Second, the framework is applicable to any NTD but it will need to be adapted for each specific NTD separately. This is because not only the program prevalence thresholds will be different, it is also expected that the spatial heterogeneity will vary across NTD. For instance, the geographical distribution of some NTD like lymphatic filariasis and schistosomiasis may be more focal (higher spatial heterogeneity) due to relatively low vector mobility as compared to, for example onchocerciasis (highly mobile vector) and STH (widespread environmental contamination). Moreover, it will be equally important for each NTD community to agree on the acceptable width of the grey zone separately for each program threshold. Third, although we focused primarily on the prevalence of STH infections of any intensity, the current framework can also be used to assess moderate-to-heavy intensity infections (WHO is striving to reduce the number of children that carry a moderate-to-heavy intensity infections to less than 2%) [32]. In this case, the presence of infections of any intensity is replaced by the presence of moderate-to-heavy intensity infections. Finally, we assumed that sensitivity and specificity do not vary across individuals. Yet, this assumption does not hold, as it is known that the sensitivity of Kato-Katz thick smear (and most other parasitological diagnostic methods) is positively related to infection intensity and is therefore different between individuals [15] as well as at the lower and upper boundary of the grey zone around a decision threshold. We believe that relaxing this assumption will impact the survey design, the sample size and the corresponding decision cut-off for any program prevalence thresholds. Therefore, future research should prioritize the expansion of the framework allowing for varying sensitivity as a function of egg counts and day-to-day variation in egg excretion. This expansion would allow for more insights into the impact of increased sampling (1 *vs*. 2 stool samples) and diagnostic effort (single *vs*. duplicate Kato-Katz thick smear) on the survey design, which in turn would provide a framework to make more evidence-based and cost-efficient recommendations.

## Conclusion

Our framework allows for the assessment and updating of M&E guidelines for NTD and product development choices. Using STH as a case study, we show that current M&E guideline may fall short severely, especially in low-endemic and post-control settings. Furthermore, specificity rather than sensitivity becomes a critical parameter to consider and sampling more clusters (≥10) may be necessary in case of considerable heterogeneity in the geographical distribution of NTD infections.

## Supporting information

S1 Table Parameterisation of the variables used to illustrate the 2-stage LQAS framework for STH control programs. ***T***: program decision prevalence threshold, ***LL***: lower limit of the grey zone, ***E***_***overrtreat***_: highest allowed probability of falsely continuing or upscaling an intervention within an implementation unit *i*, ***E***_***undertreat***_: highest allowed probability of prematurely stopping or scaling down interventions within an implementation unit *i*; ***ρ***_***i***_: intra-cluster correlation; ***se***_***d***_: sensitivity of an imperfect diagnostic test *D*; ***sp***_***d***_: specificity of an imperfect diagnostic test *D*; ***n***_***clust***_: number of clusters; ***n***_***sub***_: number of subjects per cluster. _: output variable and hence is not fixed on one value. *: The number of subjects per cluster was determined as the minimum required number of subjects that allow for adequate program decision-making (*E*_*overtreat*_ = 25%, *E*_*undertreat*_ = 5%) around a 2% program prevalence threshold. A total of 10,000 Monte Carlo simulations was used.
(DOCX)

S2 Table Summary of diagnostic methods used to illustrate the 2-stage LQAS framework for STH control programs. TPPs: target product profiles. (DOCX)

S1 Fig The relationship between the risk of under and overtreatment when deploying perfect diagnostic test. *ε*_*undertreat*_: probability of prematurely reducing interventions within an implementation unit ***i***; ***ε***_***overtreat***_: probability of falsely continuing or upscaling an intervention frequency within an implementation unit ***i***. The blue indicates the area in which the combination of the risk of under and overtreatment allow for adequate decision-making (***ε***_***undertreat***_ = **5**%, ***ε***_***overtreat***_ = **25**%). We defined the grey zone as ***T***±**50**% (**2**%: ***lower limit*** = **1**% and ***upper limit*** = **3**%). The intra-cluster correlation ***ρ***_***i***_ was set at **0.02** and the number of clusters at 5 and the number of subjects per cluster at 50. The graph was based on 10,000 Monte Carlo simulations.
(TIF)
