# Supplementary material for: Two-stage lot quality assurance sampling framework for monitoring and evaluation of neglected tropical diseases, allowing for imperfect diagnostics and spatial heterogeneity
Source: PLoS Negl Trop Dis. 2022 Apr 8;16(4):e0010353. doi: 10.1371/journal.pntd.0010353 (PMC9020685; doi:10.1371/journal.pntd.0010353)
Supplement: S1 Table — T: program decision prevalence threshold, LL: lower limit of the grey zone, Eoverrtreat: highest allowed probability of falsely continuing or upscaling an intervention within an implementation unit i, Eundertreat: highest allowed probability of prematurely stopping or scaling down interventions within an implementation unit i; ρi: intra-cluster correlation; sed: sensitivity of an imperfect diagnostic test D; spd: specificity of an imperfect diagnostic test D; nclust: number of clusters; nsub: number of subjects per cluster. _: output variable and hence is not fixed on one value. *: The number of subjects per cluster was determined as the minimum required number of subjects that allow for adequate program decision-making (Eovertreat = 25%, Eundertreat = 5%) around a 2% program prevalence threshold. A total of 10,000 Monte Carlo simulations was used. (DOCX) [file pntd.0010353.s001.docx]

**S1 Table. Parameterisation of the variables used to illustrate the 2-stage LQAS framework for STH control programs**

| $\boldsymbol{T}$ | $\boldsymbol{LL (\%)}$ | $\boldsymbol{E}_{\boldsymbol{overtreat}}$ | $\boldsymbol{UL (\%)}$ | $\boldsymbol{E}_{\boldsymbol{undertreat}}$ | $\boldsymbol{\rho}_{\boldsymbol{i}}$ | $\boldsymbol{n}_{\boldsymbol{clust}}$ | $\boldsymbol{n}_{\boldsymbol{sub}}$ | $\boldsymbol{s}\boldsymbol{e}_{\boldsymbol{d}}\boldsymbol{(\%)}$ | $\boldsymbol{s}\boldsymbol{p}_{\boldsymbol{d}}$(%) |
| --- | --- | --- | --- | --- | --- | --- | --- | --- | --- |
| The required diagnostic performance when the current WHO survey design is applied | | | | | | | | | |
| 2% | 1.6 | 0.10; 0.25 | 2.4 | 0.05 | 0.02 | 5 | 50 | 60 - 100 | 60 - 100 |
| 10% | 8 | 0.10; 0.25 | 12 | 0.05 | 0.02 | 5 | 50 | 60 - 100 | 60 - 100 |
| 20% | 16 | 0.10; 0.25 | 24 | 0.05 | 0.02 | 5 | 50 | 60 - 100 | 60 - 100 |
| 50% | 40 | 0.10; 0.25 | 60 | 0.05 | 0.02 | 5 | 50 | 60 - 100 | 60 - 100 |
| Optimise the survey design when using an imperfect test | | | | | | | | | |
| Impact of the grey zone on $\boldsymbol{n}_{\boldsymbol{sub}}$ | | | | | | | | | |
| 2% | 1.5 | 0.25 | 2.5 | 0.05 | 0.02 | 5, 10, 15, 20 | _ | 80 | 98 |
|  | 1.2 |  | 2.8 |  |  | 5, 10, 15, 20 | _ |  |  |
|  | 1 |  | 3 |  |  | 5, 10, 15, 20 | _ |  |  |
|  | 0.8 |  | 3.2 |  |  | 5, 10, 15, 20 | _ |  |  |
|  | 0.5 |  | 3.5 |  |  | 5, 10, 15, 20 | _ |  |  |
| Impact of diagnostic performance on $\boldsymbol{n}_{\boldsymbol{sub}}$ | | | | | | | | | |
| 2% | 1 | 0.25 | 3 | 0.05 | 0.02 | 5 | _ | 80 - 100 | 98 - 100 |
|  |  |  |  |  |  | 10 | _ |  |  |
|  |  |  |  |  |  | 15 | _ |  |  |
|  |  |  |  |  |  | 20 | _ |  |  |
| Impact of the geographical variation in prevalence between clusters on $\boldsymbol{n}_{\boldsymbol{sub}}$ | | | | | | | | | |
| 2% | 1 | 0.25 | 3 | 0.05 | 0.01 - 0.032 | 10 | _ | 80 | 98 |
|  |  |  |  |  |  | 15 | _ |  |  |
|  |  |  |  |  |  | 20 | _ |  |  |
|  |  |  |  |  |  |  |  |  |  |
| Customize the sample throughput and cost per test according to the improvements in diagnostic performance | | | | | | | | | |
| 2% | 1 | 0.25 | 3 | 0.05 | 0.02 | 10 | 350* | 80 | 94 |
|  |  |  |  |  |  |  | 250* | 80 | 96 |
|  |  |  |  |  |  |  | 175* | 80 | 98 |
