# Supplementary material for: Two-stage lot quality assurance sampling framework for monitoring and evaluation of neglected tropical diseases, allowing for imperfect diagnostics and spatial heterogeneity
Source: PLoS Negl Trop Dis. 2022 Apr 8;16(4):e0010353. doi: 10.1371/journal.pntd.0010353 (PMC9020685; doi:10.1371/journal.pntd.0010353)
Supplement: S2 Table — (DOCX) [file pntd.0010353.s002.docx]

**S2 Table. Summary of diagnostic methods used to illustrate the 2-stage LQAS framework for STH control programs**

| Diagnostic method name | Sensitivity (%) | Specificity (%) |
| --- | --- | --- |
| Theoretical diagnostic method | | |
| $\boldsymbol{D}_{\boldsymbol{t}\boldsymbol{1}}$ | $se_{dt1} (80)$ | $sp_{dt1}(98)$ |
| $\boldsymbol{D}_{\boldsymbol{t}\boldsymbol{2}}$ | $se_{dt2}$ (80) | $sp_{dt2}(96)$ |
| $\boldsymbol{D}_{\boldsymbol{t}\boldsymbol{3}}$ | $se_{dt3} (80)$ | $sp_{dt3}(94)$ |
| Kato-Katz thick smear | | |
| $\boldsymbol{KK}$ | $se_{kk}(55)$ | $sp_{kk}(95)$ |
| Kato-Katz thick smear with improved sensitivity | | |
| $\boldsymbol{K}\boldsymbol{K}_{\boldsymbol{se}}$ | $se_{kkse} (60)$ | $sp_{kkse} (95)$ |
| Kato-Katz thick smear with improved sensitivity | | |
| $\boldsymbol{K}\boldsymbol{K}_{\boldsymbol{sp}}$ | $se_{kksp} (55)$ | $sp_{kksp} (99)$ |
| Diagnostic methods meeting WHO TPPs | | |
| $\boldsymbol{D}_{\boldsymbol{tpp}\boldsymbol{1}}$ | $se_{dtpp1}(60)$ | $sp_{dtpp1} (99)$ |
| $\boldsymbol{D}_{\boldsymbol{tpp}\boldsymbol{2}}$ | $se_{dtpp2} (86)$ | $sp_{dtpp2} (94)$ |
