# Supplementary figures and images for: Two-stage lot quality assurance sampling framework for monitoring and evaluation of neglected tropical diseases, allowing for imperfect diagnostics and spatial heterogeneity
Source: PLoS Negl Trop Dis. 2022 Apr 8;16(4):e0010353. doi: 10.1371/journal.pntd.0010353 (PMC9020685; doi:10.1371/journal.pntd.0010353)

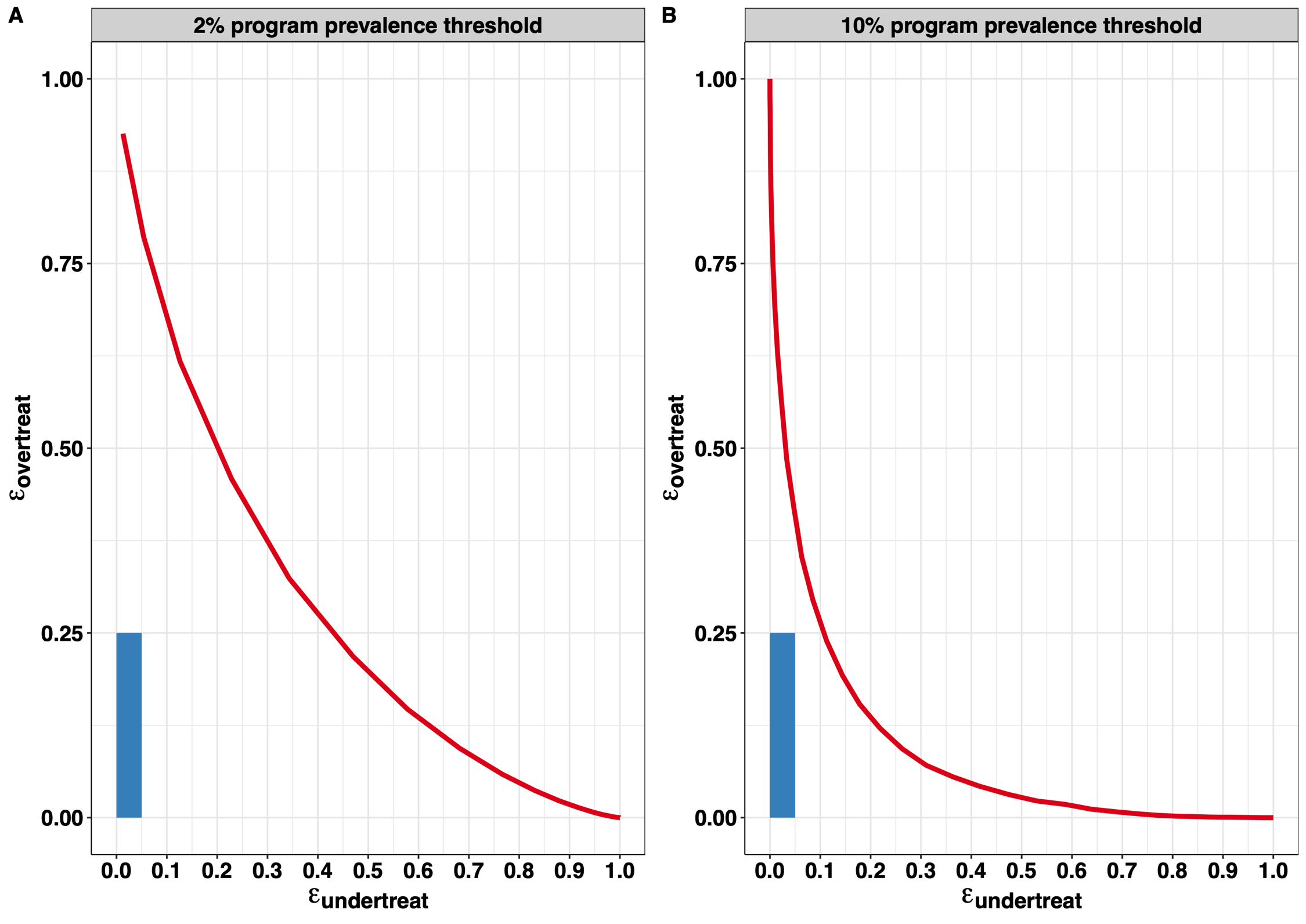

Supplement: S1 Fig — εundertreat: probability of prematurely reducing interventions within an implementation unit i; εovertreat: probability of falsely continuing or upscaling an intervention frequency within an implementation unit i. The blue indicates the area in which the combination of the risk of under and overtreatment allow for adequate decision-making (εundertreat = 5%, εovertreat = 25%). We defined the grey zone as T±50% (2%: lower limit = 1% and upper limit = 3%). The intra-cluster correlation ρi was set at 0.02 and the number of clusters at 5 and the number of subjects per cluster at 50. The graph was based on 10,000 Monte Carlo simulations. (TIF) [file pntd.0010353.s003.tif]
